# New xylose transporters support the simultaneous consumption of glucose and xylose in *Escherichia coli*

**DOI:** 10.1002/mlf2.12021

**Published:** 2022-06-10

**Authors:** Xinna Zhu, Feiyu Fan, Huanna Qiu, Mengyao Shao, Di Li, Yong Yu, Changhao Bi, Xueli Zhang

**Affiliations:** ^1^ Tianjin Institute of Industrial Biotechnology Chinese Academy of Sciences Tianjin China; ^2^ Key Laboratory of Systems Microbial Biotechnology Chinese Academy of Sciences Tianjin China; ^3^ College of Biotechnology Tianjin University of Sciences and Technology Tianjin China; ^4^ University of Chinese Academy of Sciences Beijing China

**Keywords:** ABC transporter, glucose, PTS, xylose, xylose transporter

## Abstract

Glucose and xylose are two major components of lignocellulose. Simultaneous consumption of glucose and xylose is critical for engineered microorganisms to produce fuels and chemicals from lignocellulosic biomass. Although many production limitations have been resolved, glucose‐induced inhibition of xylose transport remains a challenge. In this study, a cell growth‐based screening strategy was designed to identify xylose transporters uninhibited by glucose. The glucose pathway was genetically blocked in *Escherichia coli* so that glucose functions only as an inhibitor and cells need xylose as the carbon source for survival. Through adaptive evolution, omics analysis and reverse metabolic engineering, a new phosphoenolpyruvate: carbohydrate phosphotransferase system (PTS) galactitol transporter (GalABC, encoded by *EcolC_1640*, *EcolC_1641*, and *EcolC_1642* genes) that is not inhibited by glucose was identified. Inactivation of adenylate cyclase led to increased expression of the *EcolC_1642* gene, and a point mutation in gene *EcolC_1642* (N13S) further enhanced xylose transport. During the second round of gene mining, AraE and a new ABC transporter (AraFGH) of xylose were identified. A point mutation in the transcription regulator *araC* (L156I) caused increased expression of *araE* and *araFGH* genes without arabinose induction, and a point mutation in *araE* (D223Y) further enhanced xylose transport. These newly identified xylose transporters can support the simultaneous consumption of glucose and xylose and have potential use in producing chemicals from lignocellulose.

## INTRODUCTION

Lignocellulose is the most abundant source of biomass in the world^1^. It accounts for approximately 50% of biomass in the world, and it has been estimated that 10–50 billion tons of biomass is produced annually^2^. The main sugars in lignocellulose are glucose and xylose, which make up 30%–50% and 5%–20%, respectively, of the sugars present^3^. Thus, efficient coconsumption of glucose and xylose is significant to produce fuels and chemicals from lignocellulosic biomass.

*Escherichia coli* has been widely engineered to convert sugars to fuels and chemicals. However, xylose can be consumed by *E. coli* only after the depletion of glucose for two reasons. First, expression of genes in the xylose utilization pathway is repressed by glucose because of carbon catabolite repression (CCR), leading to a low xylose utilization rate when both glucose and xylose are the carbon sources^4^, ^5^. In *E. coli*, CCR is related to the phosphorylation status of EIIA^Glc^, which is the IIA subunit of the phosphoenolpyruvate (PEP): carbohydrate phosphotransferase system (PTS), as well as to cAMP levels. When glucose is present, the phosphate in EIIA^Glc^ is preferentially transferred to glucose during transport, generating glucose‐6‐phosphate, and dephosphorylated EIIA^Glc^ cannot activate adenylate cyclase to promote cAMP synthesis, resulting in low cAMP levels. Without cAMP, cAMP receptor protein (CRP) cannot activate the transcription of xylose catabolic operons, including *xylAB*, *xylFGH*, and *xylE* genes. This transcriptional repression problem can be solved by either inactivating the PTS system to eliminate CCR^6^, ^7^, ^8^, ^9^ or modulating the expression of key genes with artificial promoters^10^.

Xylose transport is severely inhibited in the presence of glucose. Two different xylose‐specific transport systems in *E. coli* have been characterized. One is XylE, which belongs to the major facilitator superfamily (MFS) of transporters and has a relative low‐affinity to D‐xylose with a *K*_m_ between 63 and 169 μM^11^. The other is XylFGH, which belongs to ABC family of transporters and has a high‐affinity to D‐xylose with a *K*_m_ between 0.2 and 4 μM^11^, ^12^. The expression of both *xylE* and *xylFGH* genes is repressed by the low cAMP level caused by the presence of glucose. Crp* (I112L, T127I, and A144T) mutant encoding a cAMP‐independent CRP variant and XylR* (R121C and P363S) mutant were found to improve the expression of *xylE* and *xylFGH* genes in the presence of glucose^13^, ^14^. More importantly, glucose can combine directly with XylE, blocking the binding site of xylose, and preventing xylose transport when glucose is present^15^. Although XylFGH activity is not inhibited by glucose, this transporter wastes energy during the xylose transport^12^, ^16^. Thus, simultaneous consumption of glucose and xylose in *E. coli* is difficult to achieve; therefore, it is important to discover xylose transporters that are not inhibited by glucose.

In this study, a coupled cell growth strategy was developed to identify potential xylose transporters uninhibited by glucose in *E. coli*. New xylose transporters, EcolC_1642* (N13S) belonging to the facilitated diffusion PTS family, AraE* (D233Y) belonging to the MFS, and AraFGH belonging to the ABC transporter family, were identified. All three of these xylose transporters support simultaneous consumption of glucose and xylose, which may be useful for constructing microbial cell factories to convert lignocellulosic sugars into value‐added chemicals.

## RESULTS

### Design and construction of a platform strain for screening xylose transporters not inhibited by glucose

In our previous work, all the genes in the xylose consumption pathway (*xylAB*, *tktA*, and *talB*) were simultaneously modulated with artificial promoter libraries to solve the aforementioned transcriptional repression problem^10^. Strain HQ304 (Table S1) was obtained from the promoter libraries and had significantly enhanced the activities of xylose isomerase (XylA), xylulokinase (XylB), transketolase (TktA), and transaldolase (TalB). The xylose utilization rate by strain HQ304 reached 0.8 g/gDCW·h (3‐fold higher than the ancestor strain) when xylose was the single carbon source^10^. When glucose and xylose were used as double carbon sources, the xylose consumption rate was 2‐fold higher than that of the ancestor strain, reaching 0.35 g/gDCW·h, but the xylose consumption rate was much lower than the glucose consumption rate of 0.69 g/gDCW·h (Figure S1 and Table S2). These results indicated that xylose consumption did not reach the level of glucose consumption due to a large lag in xylose consumption when both glucose and xylose were the carbon sources, although the transcriptional problem had been solved. The reason for this lag was presumed to be xylose transporter limitation or inhibition by glucose. Therefore, xylose transporters not inhibited by glucose need to be identified to promote the simultaneous consumption of glucose and xylose.

**Figure 1:**
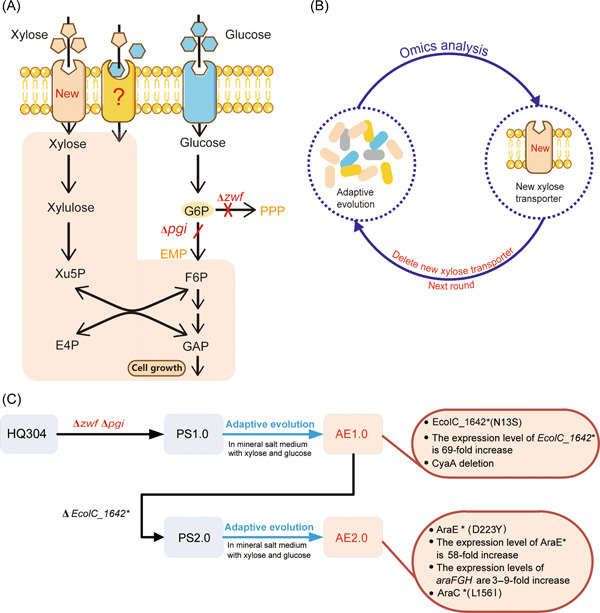
Design strategy to obtain *Escherichia coli* xylose transporters not inhibited by glucose. (A) The transport and metabolism of xylose and glucose. The pillars with different shapes on the top represent transporters, for example, blue represents glucose transporters; yellow and peachpuff represent xylose transporters. Blue hexagons and peachpuff pentagons represent glucose and xylose molecules, respectively. The red letters (*zwf* and *pgi*) and crosses represent deleted genes, which led to a disrupted glucose consumption pathway. Although glucose can be transported by glucose transporters (blue pillar), it can also bind directly with xylose transporters (yellow pillar) and block xylose transport. When the glucose pathway is interrupted, glucose cannot be metabolized, although it can be transported into a cell. In this case, cells do not grow efficiently because xylose transport is inhibited by glucose and cannot be transported into cells. E4P, erythrose 4‐phosphate; EMP, Embden–Meyerhof–Parnas; G6P, glucose‐6‐phosphate; F6P, fructose‐6‐phosphate; GAP, glyceraldehyde‐3‐phosphate; Xu5P, xylulose‐5‐phosphate; *zwf*, NADP^+^‐dependent glucose‐6‐phosphate dehydrogenase gene; and *pgi*, glucose‐6‐phosphate isomerase gene. Through adaptive evolution (B), a new xylose transporter that is uninhibited by glucose evolves to transport xylose, and it enables cell survival. More xylose transporters can be obtained by adaptive evolution after the newly evolved transporter is deleted. (C) The flowchart for screening the mutated *E. coli* strains with xylose transport. The lines with arrows represent the main steps. Two key enzymes (Zwf and Pgi) involved in glucose metabolic pathways were inactivated in HQ304 as starting strain. Adaptive evolution in minimal salt medium was used to obtain mutated strains with the ability to transport xylose. The ovals in the dark red show the main information about gene mutation loci and gene expression in metabolically evolved strains.

In this study, a cell growth‐based strategy for screening potential xylose transporters was designed (Figure 1). First, the glucose pathway was blocked, leading to disrupted glucose metabolism, suppressing growth on glucose but where glucose functioned only as an “inhibitor.” To block glucose catabolism, two key genes in the branch point of the pathway were inactivated. One is the *pgi* gene (encoding glucose‐6‐phosphate isomerase), and the other is the *zwf* gene (encoding glucose‐6‐phosphate dehydrogenase), which can convert glucose‐6‐phosphate into the Embden–Meyerhof–Parnas (EMP) pathway and pentose phosphate pathway (PPP), respectively (Figure 1A). Second, adaptative evolution was conducted to obtain cells that use a new xylose transporter to transport xylose for cell survival in glucose‐xylose mixtures in which glucose acts only as an inhibitor. Third, some omics technologies and analyses were used to identify previously undiscovered xylose transporters, and more xylose transporters were obtained through adaptive evolution after a newly evolved transporter was deleted (Figure 1B,C).

**Figure 2:**
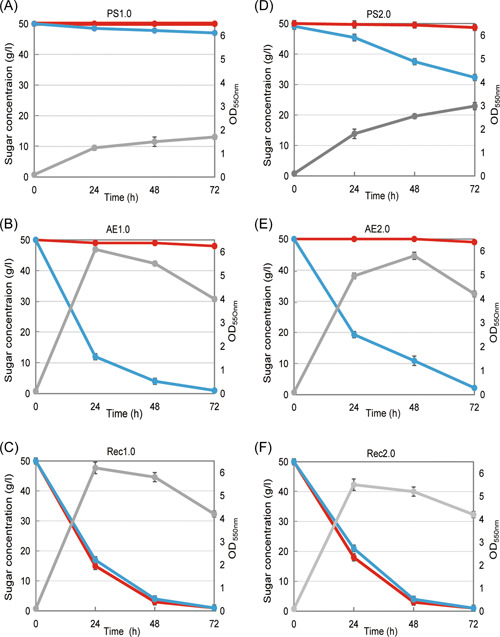
Coconsumption of glucose and xylose by different strains grown on AM1 medium. (A) Strain PS1.0, (B) Strain AE1.0, (C) Strain Rec1.0, (D) Strain PS2.0, (E) Strain AE2.0, (F) Strain Rec2.0. Assessments were performed with glucose‐xylose mixtures (50 g/l of each sugar for 72 h). Glucose and xylose concentrations and cell optical density (OD_550nm_) were determined and represented by red, blue, and gray lines, respectively. Error bars represent SD, *n* = 3.

Strain PS1.0 was obtained after knocking out the *pgi* and *zwf* genes in *E. coli* strain HQ304. Notably, in strain HQ304, the native xylose transporter XylFGH is inactivated to conserve energy during fermentation since ATP is consumed when XylFGH transports xylose^10^. When grown in a mineral salt medium with glucose and xylose, strain PS1.0 did not consume glucose as expected, and only 3 g/l xylose was consumed after 72 h (Figure 2A). The xylose consumption rate was 0.1 g/gDCW·h, and the growth rate was 0.05 h^−1^ (Table S2). Adaptive evolution was then used to improve cell growth and xylose consumption.

### Identification of primary genetic changes that accelerate xylose transport through the first round of adaptive evolution

Initially, mineral salt medium containing 30 g/l xylose and 90 g/l glucose was used for the adaptive evolution of strain PS1.0 (Figure S2A). After adaptive evolution through 34 transfers (54 days), the xylose consumption rate increased from 0.1 to 0.34 g/gDCW·h, and the growth rate increased from 0.05 to 0.23 h^−1^. Then, 50 g/l xylose and 80 g/l glucose was used for further evolution through 43 transfers (42 days) and led to the adaptively evolved strain AE1.0 (Figure 2B). Strain AE1.0 had a xylose consumption rate of 0.8 g/gDCW·h and a growth rate of 0.25 h^−1^, which increased 8‐ and 5‐fold, respectively, compared to the precursor strain, PS1.0 (Table S2).

To understand the genetic mechanisms critical for the increased xylose consumption rate, the adaptively evolved strain AE1.0, and precursor strain PS1.0 were analyzed by genome sequencing. Compared to strain PS1.0, strain AE1.0 had point mutations in the coding regions of *EcolC_1642* (encoding PTS galactitol transporter subunit IIC) and *ycdY* genes, and one 2439‐bp deletion was found in three successive genes: *yifL*, *cyaY*, and *cyaA* (Table 1). The *EcolC_1642* gene, which encodes the IIC component of the galactitol PTS system, had one point mutation in the coding region, leading to one changed amino acid, N13S (the mutated gene is designated EcolC_1642*), and the *cyaA* gene that encodes adenylate cyclase had a 1380‐bp deletion in the coding region, leading to a 460‐amino acid deletion at the C‐terminus of CyaA. *EcolC_1642* and *cyaA* genes are thought to be directly related to xylose transport.

**Table 1:** Identified mutations in the adaptively evolved strain AE1.0 and AE2.0.

Gene	Name	Protein	Altered amino acid (mutation nucleotide) site
*Mutations in strain AE1.0*		
*EcolC_1642*	‐	IIC component of the galactitol‐specific PTS system	N13S (A38G)
*EcolC_2564*	*ycdY*	Cytoplasmic chaperone TorD family protein	R161S (G481T)
*EcolC_4199*	*yifL*	Hypothetical protein, putative lipoprotein	Full deletion
*EcolC_4200*	*cyaY*	Frataxin‐like protein	Full deletion
*EcolC_4201*	*cyaA*	Adenylate cyclase	1380‐bp deletion from ATG (full length of 2574 bp)
*Mutations in strain AE2.0*			
*EcolC_0872*	*kduI*	Hexanoate isomerase	G93C (G277T)
*EcolC_0874*	*araE*	Arabinose‐proton symporter	D223Y (G667T)
*EcolC_0878*	*galR*	Transcriptional regulator	Δ72 bp/coding (23‐94/1032 nt)
*EcolC_3593*	*araC*	Transcriptional regulator, AraC family	L156I (C466A)

PTS, phosphotransferase system.

### Characterization of the physiological effects of mutations in EcolC_1642 and CyaA

To demonstrate the effects of mutations in *EcolC_1642* and *cyaA* on xylose consumption, reverse metabolic engineering was performed with precursor strain PS1.0. Replacement of the wild‐type gene *EcolC_1642* with the mutated form (N13S) in the PS1.0 chromosome increased the xylose consumption rate from 0.1 to 0.31 g/gDCW·h and the growth rate from 0.02 to 0.09 h^−1^ (Table 2). Deleting the *cyaA* gene in PS1.0 also increased the xylose consumption rate from 0.1 to 0.54 g/gDCW·h and the growth rate from 0.02 to 0.17 h^−1^ (Table 2). When introducing *EcolC_1642* and *cyaA* mutants into PS1.0 simultaneously, the xylose consumption rate and the cell growth rate increased to 0.77 g/gDCW·h and 0.24 h^−1^, which were comparable with those of the adaptively evolved strain AE1.0 with 0.8 g/gDCW·h and 0.25 h^−1^, respectively (Table 2). When the mutated *EcolC_1642* or *cyaA* was replaced by the respective wild‐type gene in AE1.0 individually or in combination, the xylose consumption rate significantly decreased to 0.49, 0.16, and 0.11 g/gDCW·h, and the cell growth rates were 0.08, 0.06 and 0.02 h^−1^ (Table 2), respectively. These results demonstrated that the enhanced xylose consumption capacity acquired during evolution was caused by the combination of the *EcolC_1642* mutation and *cyaA* deletion.

**Table 2:** Reverse metabolic engineering to demonstrate the effects of mutations on xylose utilization.

Strains^a^	Xylose utilization rate (g/gDCW·h)	Specific growth rate (h^−1^)	Cell mass (g/l)
PS1.0	0.10 ± 0.02	0.02 ± 0.00	0.14 ± 0.00
PS1.0, introducing EcolC_1642* (N13S)	0.31 ± 0.04	0.09 ± 0.01	0.73 ± 0.01
PS1.0, Δ*cyaA*	0.54 ± 0.02	0.17 ± 0.02	1.38 ± 0.01
PS1.0, introducing EcolC_1642* (N13S), Δ*cyaA*	0.77 ± 0.02	0.24 ± 0.01	1.88 ± 0.01
AE1.0	0.80 ± 0.01	0.25 ± 0.01	1.98 ± 0.01
AE1.0, recovering EcolC_1642* to wild‐type	0.49 ± 0.02	0.08 ± 0.01	0.61 ± 0.01
AE1.0, recovering *cyaA* mutation to wild‐type	0.16 ± 0.02	0.06 ± 0.00	0.45 ± 0.00
AE1.0, recovering EcolC_1642* and *cyaA* mutation to wild‐type	0.11 ± 0.01	0.02 ± 0.01	0.19 ± 0.02
PS2.0	0.16 ± 0.01	0.023 ± 0.01	0.18 ± 0.01
PS2.0, introducing araE*(D223Y)	0.40 ± 0.02	0.13 ± 0.01	1.05 ± 0.06
PS2.0, introducing araC*(L156I)	0.51 ± 0.01	0.18 ± 0.01	1.42 ± 0.02
PS2.0, introducing araE*and araC*	0.75 ± 0.02	0.21 ± 0.001	1.68 ± 0.01
AE2.0	0.82 ± 0.02	0.21 ± 0.01	1.63 ± 0.01
AE2.0, ∆araE*	0.33 ± 0.01	0.09 ± 0.00	0.74 ± 0.02
AE2.0, ∆araC*	0.38 ± 0.02	0.09 ± 0.00	0.73 ± 0.04
AE2.0, ∆araE*∆araC*	0.15 ± 0.01	0.03 ± 0.01	0.23 ± 0.11
AE2.0, ∆*araFGH*	0.56 ± 0.01	0.12 ± 0.01	0.96 ± 0.11
Rec2.0, introducing EcolC_1642*	0.84 ± 0.02	0.26 ± 0.01	2.06 ± 0.02

^a^ Fermentation was performed in AM1 with 5% glucose and 5% xlyose. Three repeats were performed and the error bars represent standard derivation.

### The effect of the *cyaA* mutant on transcription

The *cyaA* gene encodes adenylate cyclase, which catalyzes the synthesis of cyclic AMP (cAMP). cAMP is an important signaling molecule, and its concentration levels affect the expression of many genes related to sugar metabolism, especially when cAMP binds to CRP (cAMP‐receptor protein) to form the cAMP‐CRP transcriptional regulator^17^. In this study, *cyaA* deletion led to an increase in the xylose consumption rate, and it was proposed that cAMP is involved in regulating the expression of certain genes related to xylose transport. To confirm this hypothesis, reverse metabolic engineering was performed by deleting the *cyaA* gene in precursor strain PS1.0, generating strain REV1.0. REV1.0 and AE1.0 were analyzed through transcriptome sequencing. Compared to precursor strain PS1.0, the number of upregulated genes in the REV1.0 and AE1.0 strains was 175 and 245, respectively, and the number of downregulated genes was 329 and 381, respectively. These two strains shared 103 upregulated genes and 193 downregulated genes (Figure S3). Of the 103 upregulated genes in both strains, the expression level of *EcolC_1642* gene was significantly increased, by 76‐fold in the REV1.0 strain and by 59‐fold in the AE1.0 strain (Table 3). The *EcolC_1642* is in an operon, and the expression levels of three other genes, *EcolC_1640* (encoding PTS galactitol transporter subunit IIA), *EcolC_1641* (encoding PTS galactitol transporter subunit IIB), and *EcolC_1643* (encoding ribose/galactose 5‐phosphate isomerase), also increased, by 55‐, 37‐ and 68‐fold in the REV1.0 strain, respectively, and 50‐, 67‐, and 71‐fold in the AE1.0 strain, respectively (Table 3). In addition, among the 103 common upregulated genes, the transcription levels of *EcolC_1643, EcolC_1642, EcolC_1641*, and *EcolC_1640* (since they are in one operon, we currently call it as *EcolC_1643* operon) were the highest. Therefore, it was presumed that the repression of *EcolC_1643* operon expression was released upon *cyaA* deletion; however, the regulatory process is unclear and needs to be investigated.

**Table 3:** Transcriptome results of adaptively evolved strains AE1.0 and AE2.0 and reverse metabolic engineered strain REV1.0 and REV2.0.

Gene	Name	Product	Relative expression level in AE1.0 versus PS1.0	Relative expression level in REV1.0 versus PS1.0
*EcolC_1643*	‐	Ribose/galactose 5‐phosphate isomerase	71	68
*EcolC_1642*	‐	PTS galactitol transporter subunit IIC	59	76
*EcolC_1641*	‐	PTS galactitol transporter subunit IIB	67	37
*EcolC_1640*	‐	PTS galactitol transporter subunit IIA	50	55

**Gene**	**Name**	**Product**	**Relative expression level in AE2.0 versus PS2.0**	**Relative expression level in REV2.0 versus PS2.0**
*EcolC_0874*	*araE*	Arabinose‐proton symporter	58	42
*EcolC_1734*	*araF*	L‐arabinose‐binding periplasmic protein	2.3	2
*EcolC_1735*	*araG*	Arabinose import ATP‐binding protein	9	4.8
*EcolC_1736*	*araH*	Arabinose ABC transporter permease	3.9	3.5
*EcolC_3593*	*araC*	Arabinose operon regulatory protein	3.3	6.6
*EcolC_3594*	*araB*	Ribulokinase	200	117
*EcolC_3595*	*araA*	L‐arabinose isomerase	181	88
*EcolC_3596*	*araD*	L‐ribulose‐5‐phosphate‐4‐epimerase	250	74

### The cAMP‐CRP repression of *EcolC_1643* operon expression

**Figure 3:**
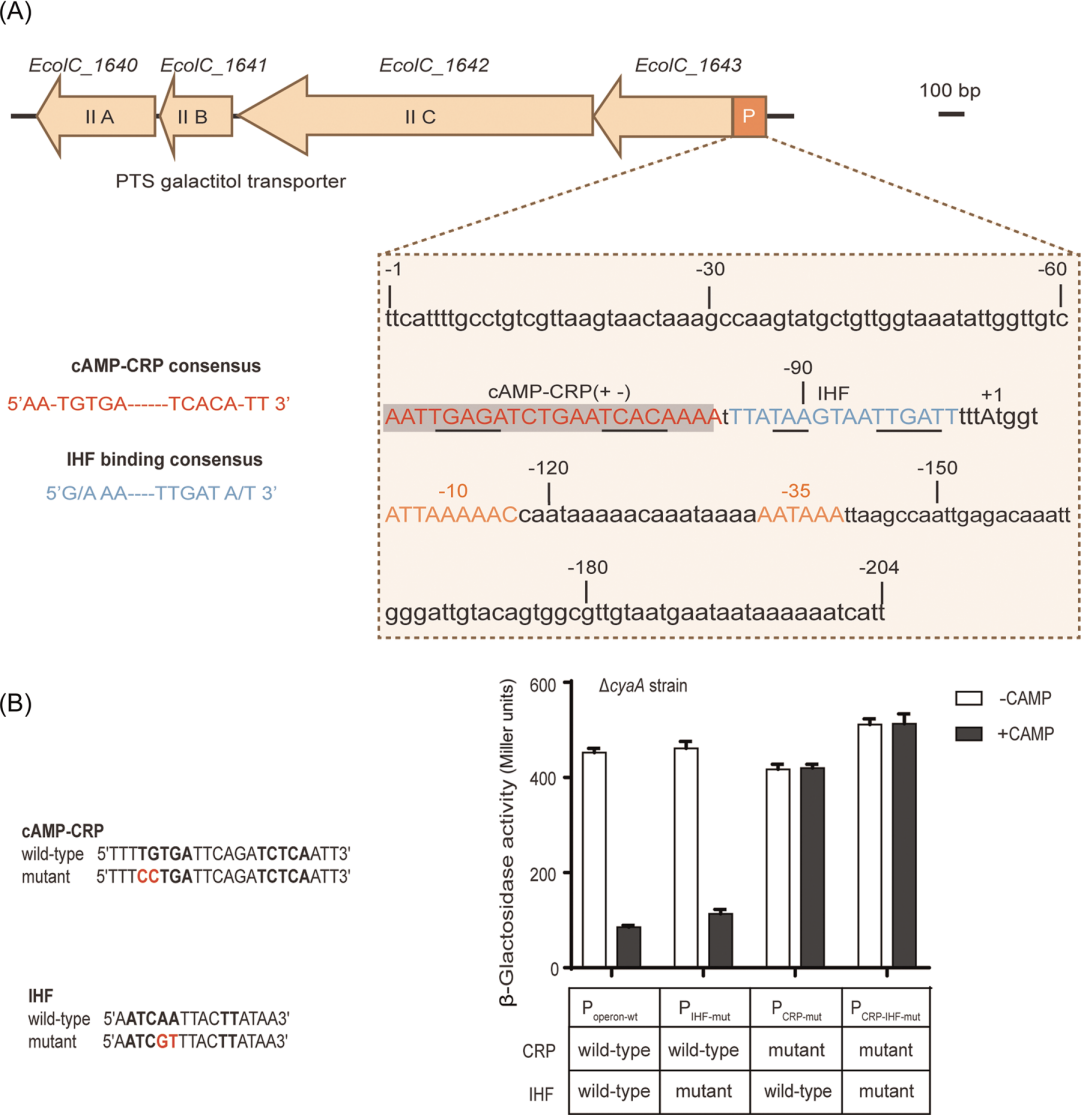
The cAMP‐CRP repression of *EcolC_1643* operon expression. (A) Schematics of the *EcolC_1643* operon and the sequence of its promoter region. The capital P represents the promoter P_operon_, the nucleotides of which are shown in the square with the dashed lines, and the promoter elements (−10 and −35 regions are in orange) and binding sites for cAMP‐CRP (in red and with shaded background) and integration host factor (IHF) (in blue) are indicated with capital letters. The underlining indicates binding sites of cAMP‐CRP or IHF, which were identified based on comparisons to the consensus sequences on the left. (B) β‐Galactosidase activity from the P_operon_‐lacZ operon mutants. For the putative binding elements of the IHF and cAMP‐CRP complex on the *EcolC_1643* operon, the wild‐type and mutant sequences are shown on the left, and the consensus sequences are in bolded black. The mutation sites are in bolded red. Three independent experiments were performed. The cells were cultured at 37°C for inducing exponential growth in the presence or absence of 2 mM cAMP. CRP, cAMP receptor protein.

To determine the reason that *cyaA* deletion affects the *EcolC_1643* operon expression, the promoter–operator region of this operon was identified and analyzed (Figure 3A). In the promoter–operator region, in addition to the −10 (ATTAAAAC) and −35 hexamer (AATAAA) region, there is a putative element (AATTGAGATCTGAATCACAAAA) for the binding of cAMP‐CRP complex at positions −62 to −83 and a putative element (TTATAAN4TTGATT) for binding of integration host factor (IHF) at positions −84 to −100. Therefore, when cAMP cannot be generated due to *cyaA* deletion, no cAMP‐CRP is formed that binds the cAMP‐CRP binding region to control expression. In addition, the IHF‐binding region can be bent^18^, which is likely an auxiliary mechanism by which cAMP‐CRP can regulate this operon's expression.

To determine whether cAMP‐CRP and IHF are involved in the regulation of this operon expression, a *ΔcyaA* strain derived from *E. coli* ATCC 8739 was constructed as a background strain to investigate the expression of P_operon_‐lacZ operon fusion on chromosomes through assessment of β‐galactosidase activity with or without cAMP. For comparison, the specific nucleotides of regulatory elements in cAMP‐CRP or IHF were changed, generating P_CRP‐mut_, P_IHF‐mut_, and P_CRP‐IHF‐mut_ (Figure 3B, left). In the absence of cAMP, the β‐galactosidase activity of P_operon‐wt_ bearing wild‐type regulatory elements was approximately 452 Miller units, while the activity significantly decreased to 85 Miller units in the presence of cAMP. The same outcome was observed with P_IHF‐mut_ bearing the wild‐type cAMP‐CRP‐binding element, and in this case, the activity decreased 4.3‐fold in the presence of cAMP, compared to the activity in the absence of cAMP. On the other hand, the β‐galactosidase activity of P_CRP‐mut_ bearing the mutation in cAMP‐CRP‐binding element was not significantly influence by cAMP, showing similar activity of approximately 417 and 420 Miller units in the presence and absence of cAMP, respectively. To determine the effects of IHF on *EcolC_1643* operon expression, the β‐galactosidase activity of P_IHF‐mut_ showed no large change compared with that of P_operon‐wt_ in the absence of cAMP, while P_IHF‐mut_ showed a small increase, of 32%, compared with P_operon‐wt_ when cAMP was added to the system. In addition, the β‐galactosidase activity of P_CRP‐IHF‐mut_ bearing both mutated elements was 6‐fold higher than that of P_operon‐wt_ in the presence of cAMP (Figure 3B, right).

**Figure 4:**
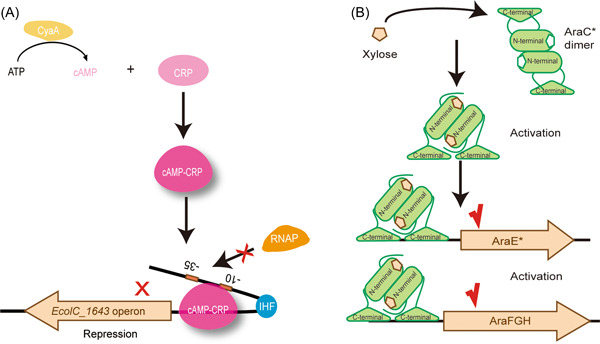
The possible regulatory model for xylose‐transporters discovered in this study. (A) Model for *EcolC_1643* operon transcriptional regulation. Elements for binding of the cAMP‐CRP complex and integration host factor (IHF) are represented by colored shapes. Promoter sequences are indicated by the small orange boxes marked −10 and −35. CyaA synthesizes cAMP, and with cAMP receptor protein (CRP), it forms the cAMP‐CRP complex. The IHF can bend the promoter sequence, enabling the −10 and −35 regions to approach the cAMP‐CRP region in space, and when cAMP‐CRP binds to this sequence, the −10 and −35 regions are blocked. As a result, RNA polymerase (RNAP) cannot access the −10 and −35 regions and the transcription of *EcolC_1643* operon is repressed. (B) The possible mechanism of AraE* and AraFGH activation by xylose‐bound AraC*. The mutant AraC* is activated by binding xylose not arabinose, and xylose‐bound AraC* is suggested to be an activator of the transcription of the *araFGH* and araE* operons.

These results indicated that cAMP‐CRP repressed the expression of this operon and that the IHF could enhance the effect of this repression. When the cAMP‐CRP complex is formed and binds to the promoter–operon region, operon expression is repressed because the smooth forward movement of RNA polymerase from the promoter region is limited. IHF functions as a spanner that can bend the DNA sequence^18^, causing the −10 and −35 regions to approach the binding element of the cAMP‐CRP complex in space. Thus, as the cAMP‐CRP complex binds the promoter operon, it blocks the −10 and −35 regions, which are key elements that RNA polymerase recognizes and binds. As a result, RNA polymerase cannot access the −10 and −35 regions, which leads to transcriptional repression of this operon. A possible model showing the transcriptional control of *EcolC_1643* operon is shown in Figure 4A.

### Identification and characterization of the new xylose transporter

The *EcolC_1640*, *EcolC_1641*, and *EcolC_1642* genes were annotated to be subunits IIA, IIB, and IIC in the galactitol‐specific PTS system (designated as GalABC). Their expression levels were significantly increased in the adaptively evolved strain AE1.0 as well as in strain REV1.0 (PS1.0 with a *cyaA* deletion mutation). Therefore, they may code for a previously unrecognized xylose transporter. To confirm this possibility, each gene including *EcolC_1640*, *EcolC_1641*, and *EcolC_1642* in the REV1.0 stain was deleted, and the resulting strains showed a significant decrease in xylose consumption rate and cell growth rate to levels similar to those of strain PS1.0 (Table S4).

The amino acid sequences of EcolC_1640, EcolC_1641, and EcolC_1642 were used as queries for domain analysis using SMART online tools. These three proteins had typical enzyme EIIA‐like (PF00359) (E‐value, 4.4e−28), EIIB‐like (PF02302) (E‐value, 6.5e−13), and EIIC‐like (PF03611) (E‐value, 4.5e−60) domains, suggesting that EcolC1640, EcolC1641 and EcolC1642 proteins compose a PTS transporter (Figure S4). In general, most PTS transporters transport various mono‐ and disaccharides, and phosphoryl groups are transferred from PTS to carbohydrates. The whole process of transferring phosphoryl groups begins with PEP and then involves EI, HPr, EIIA, and EIIB. The phosphorylated EIIB domain then phosphorylates bound sugar molecules, which are translocated to the cytosolic side of the cell membrane mediated by the EIIC domain.

EI is inactivated upon deletion of the *ptsI* gene in HQ304 (as described in previous work, HQ304 is the starting strain in this study), releasing CCR to some extent by inactivating the PTS^10^. On the other hand, inactive EI interrupts phosphoryl group transfer to sugar molecules transported by PTS transporters. Thus, we presumed that GalABC (encoded by *EcolC_1640*, *EcolC_1641*, and *EcolC_1642* genes) transport xylose in a phosphorylation‐independent manner. To confirm this hypothesis, we investigated the intracellular xylose consumption pathway to determine whether xylose is metabolized in phosphorylated form. In *E. coli*, xylose is first isomerized to xylulose by xylose isomerase (XylA), and it is then phosphorylated by xylulokinase (XylB) to become xylulose‐5‐phosphate. Xylulose‐5‐phosphate is then metabolized into the glycolysis intermediates fructose‐6‐phosphate and glyceraldehyde‐3‐phosphate. If xylose is simultaneously phosphorylated when it is transported inside the cell, it would be metabolized through a pathway independent of XylA and XylB. However, after deleting the *xylA* or *xylB* gene individually in the AE1.0 strain, cells barely grew and could not consume xylose (Table 4). Hence, it was deduced that xylose was transported inside *E. coli* cells without phosphorylation by GalABC through facilitated diffusion. This pattern of sugar molecule transportation without phosphorylation via the PTS has also been reported for galactose via the glucose PTS in *E. coli* ^19^, galactose and trehalose via the mannose PTS in *Salmonella typhimurium* ^20^, ^21^ and xylose via the mannose PTS in *Lactobacillus casei* ^22^.

**Table 4:** The effects of *xylA* and *xylB* gene deletion on xylose utilization.

Strain^a^	Xylose utilization rate (g/gDCW·h)	Specific growth rate (h^−1^)	Cell mass (g/l)
AE1.0	0.80 ± 0.01	0.25 ± 0.01	1.98 ± 0.01
AE1.0, ∆*xylA*	0.07 ± 0.004	0.00 ± 0.00	0.02 ± 0.01
AE1.0, ∆*xylB*	0.05 ± 0.005	0.00 ± 0.00	0.02 ± 0.01

^a^ Fermentation was performed in AM1 with 5% glucose and 5% xlyose. Three repeats were performed and the error bars represent standard derivation.

### Identification of a second new xylose transporter

To identify more new xylose transporters, the previously identified xylose transporter was inactivated, and another round of adaptive evolution was performed. The EcolC_1642* gene was deleted in AE1.0 to generate strain PS2.0, and the xylose consumption rate significantly decreased to 0.16 g/gDCW·h (Figure 2D). Adaptive evolution of strain PS2.0 was performed in mineral salt medium containing 50 g/l xylose and 80 g/l glucose (Figure S2B). After evolution for 38 generations (39 days), the second‐round strain AE2.0 was obtained. The xylose consumption rate and cell growth rate were 0.82 g/gDCW·h and 0.21 h^−1^, which increased 5‐ and 9‐fold compared to those of precursor strain PS2.0, respectively (Table 2 and Figure 2E).

To identify the genetic mechanisms critical for the increased xylose consumption rate, genome sequencing was performed with the strains AE2.0 and PS2.0. Four nonsilent point mutations were found in the *araE*, *araC*, and *kduI* genes in the evolved strain AE2.0 compared with precursor strain PS2.0 (Table 1). There was also a 72‐bp deletion in the coding region of the *galR* gene. Among these mutated genes, the *araE* and *araC* genes are thought to be directly related to xylose transportation. AraE is an arabinose‐proton symporter, and one point mutation in the coding region of the *araE* (G667T) gene leads to the change in one amino acid, D223Y. AraC is an arabinose operon regulatory protein, and one point mutation in the coding region of the *araC* (C466A) gene leads to one changed amino acid, L156I.

To demonstrate the effects of these two mutations on xylose utilization, reverse metabolic engineering was performed with precursor strain PS2.0. AraE (D223Y) and AraC (L156I) mutations were introduced into PS2.0 individually, and the xylose consumption rate increased from 0.16 to 0.40 and 0.51 g/gDCW·h, and the growth rate increased from 0.023 to 0.13 and 0.18 h^−1^, respectively (Table 2). After introducing both mutations into PS2.0, the xylose utilization rate and the growth rate increased to 0.75 g/gDCW·h and 0.21 h^−1^, respectively (Table 2), which were comparable to those of strain AE2.0. Replacement of the *araE* and *araC* mutants in evolved strain AE2.0 individually or in combination significantly decreased the xylose consumption rate and cell growth rate (Table 2). These results demonstrated that the increase in xylose consumption during the second round of adaptive evolution was directly related to the *araE* and *araC* mutations (designated araE* and araC*).

The gene expression levels of strain AE2.0 were also analyzed by transcriptome sequencing, and it was found that the expression levels of the araE* and araC* genes increased 58‐ and 3.3‐fold, respectively, compared with those in precursor strain PS2.0 (Table 3). The gene operons *araFGH* and *araBAD*, which are involved in the transport and metabolism of arabinose, also showed much higher expression levels in evolved strain AE2.0 (Table 3). We presumed that the mutant araC* increased the expression levels of genes related to arabinose catabolism and transport. To confirm this presumption, the araC* mutant was introduced into precursor strain PS2.0 to generate the metabolically reverse‐engineered strain REV2.0, and further transcriptome sequencing was performed. Compared to those in strain PS2.0, the expression levels of the *araE*, *araFGH*, and *araBAD* genes showed a 2‐ to 117‐fold increase in strain REV2.0 (Table 3). Further, the expression levels of *araC*, *araE*, *araFGH*, and *araBAD* genes in the first round were rechecked, and we found that they were downregulated in strain AE1.0 (Table S3). The main reason is that *cyaA* was inactive in the first round, which led to downregulation of *araC* due to cAMP‐CRP functioning as a positive regulator for *araC* expression^23^. The mutated AraC* (L156I) in the second round activated the expression levels of itself and *araE*, *araFGH*, and *araBAD* genes, consistent with AraC reported role as a transcriptional activator of these genes^24^. Next, to investigate whether the *araFGH* and *araBAD* operons are necessary for the function of xylose transport and metabolism, these two operons were deleted individually in strain PS2.0 into which the araC* mutant was introduced. For the strain with the *araFGH* gene deletion, xylose consumption decreased by 24%, from 0.51 to 0.39 g/gDCW·h, but there was no change to the strain with *araBAD* gene deletion, indicating that AraFGH contributed partially to xylose transport.

For the second round of selection, the results indicated that both AraE* and AraFGH were critical for improved xylose utilization in evolved strain Rec2.0 (Table S2). When EcolC_1642* gene obtained in the first round was introducing into AE2.0, the xylose consumption increased from 0.66 to 0.84 g/gDCW·h (Table 2), indicating that combined transporters had a synergistic effect on xylose transport.

### Simultaneous consumption of glucose and xylose

To determine whether the newly identified xylose transporters can support simultaneous consumption of glucose and xylose, the *zwf* and *pgi* genes were reestablished in strains AE1.0 and AE2.0, and these recovered strains are called Rec1.0 and Rec2.0, respectively. Both strains could simultaneously consume glucose and xylose at nearly the same rate (Figure 2C,F). The glucose consumption rates in strains Rec1.0 and Rec2.0 were 0.71 and 0.76 g/gDCW·h, respectively; the xylose consumption rates were 0.63 and 0.66 g/gDCW·h, respectively; and the ratios of sugar consumption rates were 0.94 and 0.95 mol_glucose_/mol_xylose_, nearly approaching to 1 mol_glucose_/mol_xylose_, which indicated efficient glucose and xylose coconsumption.

To resolve the problem of simultaneously consuming glucose and xylose, previous work focused on eliminating CCR and the strategies were applied to remove repression by deletion of *ptsI*, *ptsG*, and *crr* genes and mutation of *crp* and *xylR* genes^4^, ^9^, ^14^, ^25^, ^26^. However, the coutilization efficiency of glucose and xylose was still low and unbalanced (Table S5). This study mainly focused xylose transport, which is another limiting factor of low xylose consumption rate in mixed sugar fermentations. The new xylose transporters uninhibited by glucose were identified, which could support simultaneous consumption of glucose and xylose.

## DISCUSSION

### Xylose transport by PTS transporters

**Figure 5:**
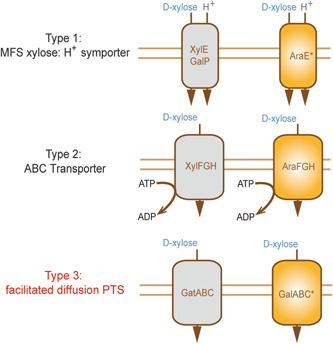
A graphic showing three types of xylose transporters identified thus far. Type 1 is MFS xylose, a H^+^ symporter with XylE, GalP and AraE; Type 2 is an ABC transporter, including XylFGH and AraFGH, which needs to consume ATP when xylose is transported; Type 3 is a facilitated diffusion PTS, including GatABC and GalABC (encoded by *EcolC_1640*, *EcolC_1641* and *EcolC_1642* genes). GalABC* with EcolC_1642*, AraE* and AraFGH are newly identified xylose transporters that are uninhibited by glucose.

There are two commonly known xylose transporter types, and they belong to MFS and ABC family. The newly identified xylose transporter GalABC (putative galactitol‐specific PTS enzymes, encoded by *EcolC_1640*, *EcolC_1641, and EcolC_1642* genes) belongs to a third family, the PTS transporter family (Figure 5). GatABC (galactitol‐specific PTS enzymes, encoded by *gatA*, *gatB*, and *gatC* genes) was the first discovered PTS transporter and was reported to be able to transport xylose when GatC is mutated (S184L, designated GatC*)^27^. However, GatABC* also demonstrated the ability to transport xylose when xylose is the single carbon source^27^, and it is not clear whether xylose can be transported when glucose and xylose are both available sources. To test whether GatABC* is inhibited by glucose, GatC* was introduced into strain PS1.0, and the gatABC* gene operon was further modulated with a strong promoter to increase its expression level. When the medium contained both glucose and xylose, this mutant strain showed little improvement in xylose consumption (Table S6). Thus, GatABC* was inhibited by glucose. In contrast, after enhancing the expression levels of the GalABC* operon (encoded by *EcolC_1640*, *EcolC_1641*, and EcolC_1642* genes) in strain PS1.0, the resulting mutant strain showed significantly increased xylose consumption when glucose and xylose were both carbon sources (Table S5). Thus, GalABC* is the first PTS transporter, which is able to transport xylose without inhibition by glucose.

There is 31% identity of amino acid sequence between GatC and EcolC_1642 in *E. coli* ATCC 8739. The *EcolC_1642* gene is not present in all Enterobacteriaceae. No *EcolC_1642* gene is present in *E. coli* MG1655, which is a model *E. coli* strain, while *Shigella*, *Klebsiella*, and *Salmonella* have genes that are similar to *Ecol_1642* (Figure S6). The *Ecol_1642* genes in different species may be useful for discovering more xylose transporters that are not inhibited by glucose.

### Homology modeling and structural analysis of EcolC_1642*

**Figure 6:**
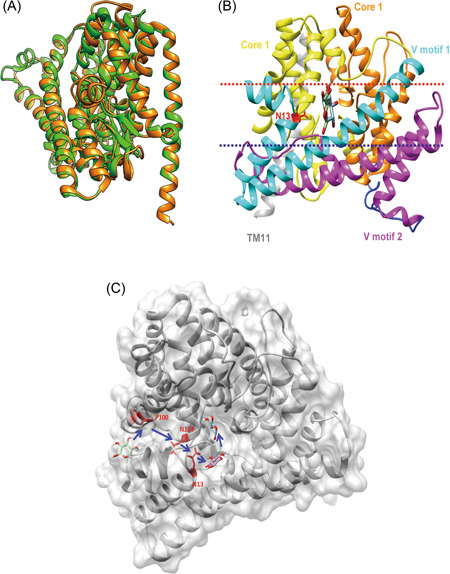
Homology model of the EcolC_1642 structure. (A) Image of the EcolC_1642 model superimposed on the out‐facing crystal structure of *E. coli* UlaA (PDB ID code 4rp8). (B) Side view of EcolC_1642 crossing the cell membrane based on a working model of the transport mechanism of UlaA^28^. The red and blue dashes show the approximate location of the cell membrane. Amino acid residue N13 (red) is located in the wing of V motif 1 (cyan). Xylose is shown in a ball‐and‐stick representation. (C) The translocation channel is shown by short blue arrows, and the amino acid residues N13, P100, and N124 on the translocation channel are shown in red.

The transcription of the EcolC_1642* gene in strain HQ408 (strain PS1.0 with introduced EcolC_1642* gene) was analyzed by quantitative polymerase chain reaction (qPCR), and the results showed no differences with that of strain PS1.0 (data not shown). Therefore, it was presumed that the N13S mutation in EcolC_1642 increased xylose transport capability but not its transcription rate. To look over the spatial position of N13 and its orientation toward bound sugar, a model homologous to EcolC_1642 (Figure 6A) was constructed using the I‐TASSER server and refined it with the GalaxyRefine program (Rama favored, 91.6% and poor rotamers, 0.8). We found that EcolC_1642 is structurally similar to the well‐characterized ascorbate‐specific permease IIC component UlaA (PDB ID code 4rp8) of *E. coli*, which has 92.5% overall structural coverage albeit with a low amino acid sequence identity of 13.5%.

Similar to the structure of UlaA^28^, EcolC_1642 consists of 11 transmembrane segments (TMs), four hairpin structures (HPs), and three horizontal amphipathic helix segments (AHs), which are spatially organized into V motif 1 (cyan), core 1 (yellow), V motif 2 (magenta), core 2 (orange), and TM11 (gray) (Figure 6B). The first two helices in each TM2 and TM6‐7 give rise to V‐shaped motifs (V motifs 1 and 2, cyan and magenta, respectively). The other TMs and HPs form transporter cores (cores 1 and 2, yellow and orange, respectively). These two core subdomains, together with TM11, form the translocation pocket domain, where the substrate xylose is located (Figure 6B). The N13S (red) is near the end of V‐motif 1.

A possible mechanism by which EcolC_1642* transports xylose without glucose inhibition was developed. Xylose transport via EcolC_1642 protein involves a translocation channel facing the translocation pocket, but amino acid residues such as asparagine (N) in the translocation channel show more affinity to glucose than to xylose, making xylose difficult to transport in the presence of glucose. However, residues such as serine (S) have the opposite effect. To obtain support for this hypothesis, some residues, including the P100 and N124 residues, in the translocation channel were replaced with serine (Figure 6C). The resulting mutants P100S and N124S showed enhanced xylose consumption rates from 0.1 to 0.27 and 0.23 g/gDCW·h, respectively (Table S7). Furthermore, combining these two mutations with N13S separately led to higher xylose consumption rates (Table S7). The best combination (P100S/N13S) had a xylose consumption rate of 0.36 g/gDCW·h, which was 3.6‐fold higher than that of precursor stain PS1.0 (Table S7). These results demonstrated that amino acid residues in the translocation channel are pivotal for xylose transport; for example, the presence of serine dramatically abolished glucose inhibition.

### Xylose transport by AraE* and AraFGH

AraE and AraFGH are arabinose transporters belonging to the arabinose/H^+^ symporter and ABC transporter families, respectively (Figure 5). Generally, AraE contributes to arabinose transport in the presence of arabinose, which is an inducer that can activate AraC protein to control the *araE* gene expression^16^. However, when xylose is used as the single carbon source, AraE can transport xylose to support cell growth^16^. Additionally, AraE in *Bacillus subtilis* was required to transport xylose because there is no specific transporter for xylose in this species^29^.

In the present work, the AraC* mutant (L156I) led to a 42‐fold increase in *araE* gene expression level (Table 3). In addition to the increased expression of the *araE*, the *araE* mutation (D223Y) also contributed to xylose transport because this mutation had no effect on the transcription level of the araE* (data not shown). The model structure of AraE in the UniProt online database (https://www.uniport.org/uniport/P0AE24) shows that the D223Y position is located in the cytoplasmic loop between the 6th and 7th transmembrane helices (Figure S5). The crystal structure of AraE binding a xylose molecule has not been resolved, and thus it is difficult to determine the true relationship between the D223Y mutation and a xylose molecule. The mechanism of improved xylose transport seen in the AraE* (D223Y) mutant will be investigated in future studies.

In addition to activating *araE* gene expression, the AraC* (L156I) activates *araFGH* gene expression. Deletion of the araE* gene in strain AE2.0 decreased the xylose consumption rate from 0.82 to 0.33 g/gDCW·h (Table 2), and after the *araFGH* gene was deleted, the xylose consumption rate decreased to 0.56 g/gDCW·h (Table 2). All these results demonstrate that AraFGH is a xylose transporter, which is reported here for the first time (Figure 5). AraE* is the main xylose transporter, contributing 75% of the xylose flux through transportation, while AraFGH is the auxiliary transporter, contributing 25% to xylose flux (Figure S7).

The regulatory protein AraC forms dimers in a cell, and its structure changes in the presence of arabinose. When arabinose is absent, a β‐barrel dimer of AraC is formed, which represses the transcription of several operons related to the transport and catabolism of arabinose. When arabinose binds to AraC, the protein dimer structure is altered, and an antiparallel coiled‐coil dimer is formed, which activates transcription of the *araE*, *araFGH*, and *araBAD* operons because the AraC binding preference is changed on the basis of binding‐site spacing^30^. In this study, the mutation (L156I) of *araC* led to activation of *araE* and *araFGH* gene expression, possibly because an antiparallel coiled‐coil AraC dimer was formed that binds xylose not arabinose. A possible regulatory model for AraC* is shown in Figure 4B, and further experiments to investigate the binding xylose to AraC* will be performed in the future.

### Inactivated CyaA is not an issue for bioconversion of lignocellulose to value‐added chemicals

cAMP‐CRP is a global regulator that involves many genes related to sugar metabolism. The evolved strain with improved xylose utilization was not able to form cAMP‐CRP due to the deletion of a portion of *cyaA*. This change might also affect the expression of other genes. We reviewed the gene expression levels in the evolved strains (AE1.0 and AE2.0), to see if the inactive CyaA had effects on the expression of genes in the main pathways (Table S8). We found that most of genes in glycolysis pathway had higher expression levels in the evolved strains than that in the parent stain (PS1.0), except for *galP* and *glk* genes. For the *galP* gene, the relative expression levels in strain AE1.0 and AE2.0 decreased by almost one half, but the absolute expression levels were still high with fragments per kilobase per million (FPKM) about 10,000. For the *glk* gene, the expression levels decreased one hundred times. When wild‐type *pgi* and *zwf* genes were introduced into AE1.0 and AE2.0, the glucose utilization rates were 0.71 and 0.76 g/gDCW·h (Table S5), indicating that the low expression of *glk* gene did not affect glucose metabolism, and possibly due to high Glk enzyme activity. On another hand, most of the genes related to the metabolism of other sugars such as galactose, tagatose, galactitol, maltose, and mannose that are regulated by cAMP‐CRP were downregulated. The main components of lignocellulose hydrolysis are glucose and xylose. Therefore, inactivation of *cyaA* would not be an issue for bioconversion of lignocellulose to value‐added chemicals using the evolved strains with mineral salt medium under anaerobic conditions.

## MATERIALS AND METHODS

### Plasmids, strains, and growth conditions

The plasmids and primers used for *zwf*, *pgi*, and *cyaA* genes deletion, and EcolC_1642* mutant integration are summarized in Supporting Information: Table S1. Strain HQ304 with high xylose utilization was constructed in our previous work^10^ and other strains were constructed in this study that are listed in Supporting Information: Table S1. During construction, strains were cultured aerobically at 30°C or 37°C in Luria broth (LB) (10 g/l Difco tryptone, 10 g/l NaCl and 5 g/l Difco yeast extract). Antibiotics such as kanamycin (50 mg/l), ampicillin (100 mg/l), or chloramphenicol (34 mg/l) were used where appropriate.

### Genetic methods

The CRISPR/Cas9 gene‐editing technique based on the two‐plasmid system (Cas9 protein and λ‐Red on the facilitated plasmid; gRNA and donor DNA on the editing plasmid) was used to delete *zwf, pgi*, or *cyaA* genes on chromosome. The facilitated plasmid pRedCas9^10^ and the editing plasmid pV4‐del‐gene (Table S1) were cotransformed into the electrophoresis competent cells, and subsequently the transformants were grown on LB plate with kanamycin and chloramphenicol at 30°C. The target gene was edited when Cas9 and λ‐Red was induced by adding L (+)‐arabinose (1% final concentration) to the culture. Additional details are included in Text S1.

### Adaptive evolution to select for a xylose transporter not inhibited by glucose

For the first‐round adaptive evolution, strain PS1.0 was consecutively transferred into AM1 mineral salt media using pH‐controlled vessels (500 ml), and working volume was a 300 ml with 37°C and 150 rpm under anaerobic condition^31^. We used 33 mgDCW/l (about 0.1 as OD_550nm_) as the beginning cell densities for each transfer. Medium was 30 g/l xylose and 90 g/l glucose for early 34 transfers (54 days) and 50 g/l xylose and 80 g/l glucose for further 43 transfers (42 days). For the second‐round adaptive evolution, medium was 50 g/l xylose and 80 g/l glucose for 40 transfers (40 days). Eight clones from the last transfer culture were selected and assessed using AM1 with 50 g/l xylose and 80 g/l glucose. Most of their phenotypes were comparable to the evolved populations. One representative clone was selected for genome sequencing.

### Genome resequencing and identification of mutational events

The total genomic DNA of each strain was extracted using the Promega Wizard genomic DNA purification kit. DNA quality was determined using a NanoDrop spectrophotometer ND‐1000 (NanoDrop Technologie). Genome sequencing of strain was commercially done by Novogene Biotechnology Co., Ltd., and two 150 bp paired‐end libraries were sequenced on an Illumina HiSeq X Ten instrument. Afterward, reads were mapped to *E. coli* ATCCT 8739 genome (GenBank CP000946.1) using Breseq pipline (v0.24)^32^, genetic variation events including single‐nucleotide mutations, small insertion and deletion (indel), large‐scale chromosomal changes, and new junction evidence were obtained and function for these mutations was selectively validated in *E. coli* system.

### Transcriptome sequencing

Cells grown in mixed sugar medium were collected at the mid‐exponential phase and incubated with two volumes of RNAprotect. Bacteria Reagent (Qiagen) was used to minimize RNA degradation. Total RNA was isolated using RNeasy Mini kit (Qiagen). The high‐throughput mRNA sequencing (2 × 150 bp paired‐end sequencing) was performed commercially at Novogene Biotechnology Co., Ltd. by using Illumina's Hiseq X Ten platform (Illumina). Quality control of raw sequencing reads was assessed by NGS QC Toolkit (version 2.3.3)^33^, and the quality trimmed and filtered reads were aligned to the genome of *E. coli* ATCC 8739 strain (GenBank CP000946.1) using the Bowtie 2 v2.2.5^34^. The unambiguously aligned reads to each gene were then tailed with HTSeq‐count scripts (0.6.0) using the intersection‐nonempty resolution mode^35^. The normalized abundance for each transcript was calculated using the FPKM mapped method^36^. Differentially expressed genes were identified using |log_2_ Ratio | ≥ 1 as the final cut‐off.

### Homology modeling and structural analysis

Initial models for EcolC_1642 were generated by I‐TASSER server (https://zhanglab.ccmb.med.umich.edu/I‐TASSER/)^37^, using the PSIPRED program^38^ to help validate the secondary structure. Subsequently, the top‐scoring model was submitted to the GalaxyRefine server (http://galaxy.seoklab.org/index.html)^39^ for rebuilding and repacking the side chain as well as suffering an overall structure relaxation by molecular dynamics simulation. Molecular docking was performed using Autodock Vina software (v1.1.2)^40^. Ligand D‐xylose and D‐glucose structure were retrieved from the ZINC site (http://zinc.docking.org/)^41^. Proteins and ligand structures were prepared for docking by using Autodock Tools v1.5.4 (grid box was set to 20 × 20 × 20 Å). All of the structures were visualized and manipulated using UCSF Chimera (v1.13.1)^42^.

### Other analytical procedures

The optical density at 550 nm (OD_550nm_) that represented biomass concentration was measured using an SP‐723 spectrophotometer (Spectrum SHANGHAI, China). Dry cell weight (DCW) was calculated by converting the OD_550nm_ values using a predetermined equation: 1 OD_550nm_ = 0.33 gDCW/l.

### β‐Galactosidase activity

β‐Galactosidase activity was detected as described by Miller^43^. Cells having P_operon_‐lacZ operon fusion were first cultured at 37°C for 12 h in 2 ml of AM1 medium with 10 g/l glucose and then 20 µl of the preculture was inoculated into 2 ml AM1 medium with 10 g/l glucose, incubated at 37°C with shaking. 2 mM cAMP was added as required. 1 ml of cells was collected by centrifugation, and the cell pellets were washed and resuspended in phosphate‐buffered saline (PBS), and then the optical density at 600 nm was measured. The reaction of β‐galactosidase assays was measured by optical density at 420 nm, and the activity of Miller units was calculated as below: activity (*U*) = 1000 × A420/[time (min) × A600 × vol (ml)].

## AUTHOR CONTRIBUTIONS

Xueli Zhang and Changhao Bi designed research; Mengyao Shao, Yong Yu, Di Li, and Huanna Qiu performed research; Xinna Zhu and Feiyu Fan analyzed data; and Xinna Zhu and Xueli Zhang wrote the paper.

## ETHICS STATEMENT

There is no animal used in this study.

## CONFLICT OF INTERESTS

This study has been included in patent applications by the Tianjin Institute of Industrial Biotechnology, Chinese Academy of Sciences.

## Supporting information

Supplementary information.

Supplementary information.

## Data Availability

Data supporting the findings of this study are available within the paper or from the corresponding author upon request. Request for materials should be addressed to the corresponding author. The sequences reported in this paper have been deposited in NCBI's Gene Expression database (accession no. GSE186804).
