# Supplementary material for: New xylose transporters support the simultaneous consumption of glucose and xylose in Escherichia coli
Source: mLife. 2022 Jun 10;1(2):156–70. doi: 10.1002/mlf2.12021 (PMC10989795; doi:10.1002/mlf2.12021)
Supplement: Supplementary file 2 — Supplementary information. [file MLF2-1-156-s001.docx]

**Supporting Information**

**Text S1**

**Gene Editing Using a Two-plasmid based CRISPR/Cas9 method.** In previous work (Yu et al., 2020), we described a two-plasmid based CRISPR/Cas9 method for consecutive gene editing. pRedCas9 as an auxiliary plasmid was for recombination and cleaving, and donor plasmid was to express the gRNA, homologous fragments or integration fragment.

For the deletion of the *zwf* gene in *E. coli*, the donor plasmid pV4-del-zwf was constructed by inserting the zwf-N20-gRNA fragment and donor DNA fragment into plasmid pV4 (Table S1). The primer pair N20-B-F1/N20-B-R1 was used to amplify the fragment of the pV4 backbone, including the elements *cat*, P15A and lacI-Ptrc-cat-N20-gRNA. Using the same pV4 as template, the N20 sequence targeting the *zwf* gene was amplified using the primer pair zwf-N20-B-F2/N20-B-R2, and zwf-N20 was included in the forward primer. Further, the donor DNA for zwf gene deletion was prepared using primer sets zwf-F1/zwf-R1 and zwf-F2/zwf-R2, obtaining up-stream and down-stream homologous sequences, respectively. These three PCR fragments were assembled by Golden Gate technology (Engler et al., 2008), resulting in plasmid pV4-del-zwf. The donor plasmid including pV4-del-pgi and pV4-del-cyaA for *pgi* and *cyaA* genes deletions were prepared in the same way (Table S1). Primers used are also listed in Table S1.

**Modulation and Deletion of Genes by λ-Red recombination system.** The **λ-**Red recombination system was used to modulate and deletion some genes. First, a two-step homologous recombination method (Chen et al., 2014) was used to modulate *EcolC_1642** operon and *gatABC** operon with artificial regulatory part M1-93 (Lu et al., 2012), which strength is 5-times of induced *E. coli* *lacZ* promoter. Second, a one-step homologous recombination method was used to delete *EcolC_1640*, *EcolC_1641* and *EcolC_1642* genes, and *araFGH* and *araBDA* operons as described by Datsenko (Datsenka 2001).

**Integration of *P_operon_*-lacZ Operon Fusion into the Genome with ΔcyaA Background**. The *cyaA* gene was deleted in *E. coli* ATCC 8739 to generate the ΔcyaA strain. This strain was used to construct a *P_operon_*-lacZ operon by changing the native promoter of *lacZ* gene to promoter-operater of EcolC_1643 operon.

The DNA fragment including the promoter-operator region of *EcolC_1643* operon (corresponding to positions 204 to 1 in Fig. 3A) was amplified by PCR with the primer sets P_operon_-up/P_operon_-down and *E. coli* ATCC 8739 genomic DNA as a template (Zhang et al., 2009). The amplified fragment was inserted into the pEASY-Blunt vector (Beijing TransGen Biotech Co., Ltd) to generated pP_operon_-WT.

Site-directed mutagenesis was performed for introducing site-specific mutations into the putative cAMP-CRP element and IHF element. Using pP_operon_-WT as template, the mutagenic primer sets CRP-mut-up/CRP-mut-down, IHF-mut-up/IHF-mut-down, CRP-IHF-mut-up/CRP-IHF-mut-up, were performed for construction plasmids containing mutations of cAMP-CRP- and IHF- binding elements individually or in combined, generating pP_operon_-CRP, pP_operon_-IHF, and pP_operon_-CRP-IHF.

The native promoter (122 bp) of *lacZ* in the ΔcyaA strain was replaced by the promoter-operator (204 bp) of *EcolC_1643* operon through two-step recombination methods (Tan et al., 2014). In the first recombination, *cat-sacB* cassette was amplified with primer set lacZ-cat-up/lacZ-sacB-down and pXZ-CS as template and used to replace *lacZ* promoter in the ΔcyaA strain. In the second recombination, the promoter-operater of *EcolC_1643* operon including wild-type and mutations were amplified with a primer set P_operon_-lacZ-up/P_operon_-lacZ-down and pP_operon-_WT, pP_operon_-CRP, pP_operon_-IHF, and pP_operon_-CRP-IHF as templates and used to replace *cat-sacB* cassette by selection for resistance to sucrose. Cells containing *sacB* gene accumulate levan during incubation with sucrose and are killed. Surviving recombinants are highly enriched for loss of cat-sacB cassette (Jantama et al., 2008; Zhang et al., 2007).

**Costruction of EcolC_1642 Mutations.** The DNA fragmenet including wild-type *EcolC_1642* gene and mutate *EcolC_1642**(N13S) gene were amplified by PCR with the primer sets 1642-CX-up/1642-CX-down, and *E. coli* ATCC 8739 genomic DNA and strain AE1.0 DNA as a template, respectively. The amplified fragment was inserted into the pEASY-Blunt vector (Beijing TransGen Biotech Co., Ltd) to generated pEASY-1642-WT and pEASY-1642-N13S. To generate pEASY-1642-P100S, pEASY-1642-N124S with single mutation, and pEASY-1642-P100S/N13S, pEASY-1642-N124S/N13S with combined mutation, site-directed mutagenesis was used as described above. by desinating mutations on primers

These mutantions *EcolC_1642* gene including single muatation P100S, N124S and combined mutation P100S/N13S, N124/N13S were integrated into strain PS1.0 to replace the wild-type *EcolC_1642* gene using two-step homologous recombination as described above. Generating strain P100S, N124S, P100S/N13S, and N124S/N13S.

**Co-consumption Assessment.** Co-consumption were assessed using AM1 mineral salt media supplemented with glucose and xylose at each concentration of 50 g·L^-1^. Seed culture were grown overnight (16 h) in AM1 medium supplemented with 2% sugar. For each experiment, *E. coli* was grown at 37 ℃ in a pH-controlled fermentation vessel with the initial cell density at OD_550nm_ of 0.1 as previously described (Zhu et al., 2014). Glucose and xylose were measured by HPLC.

**Figure S**

Fig. S1. Co-utilization of glucose-xylose mixtures for the strain HQ304 using AM1 medium. 50 g/L of each sugar as initial concentration, The data points represent averages and standard deviations of three replicates.


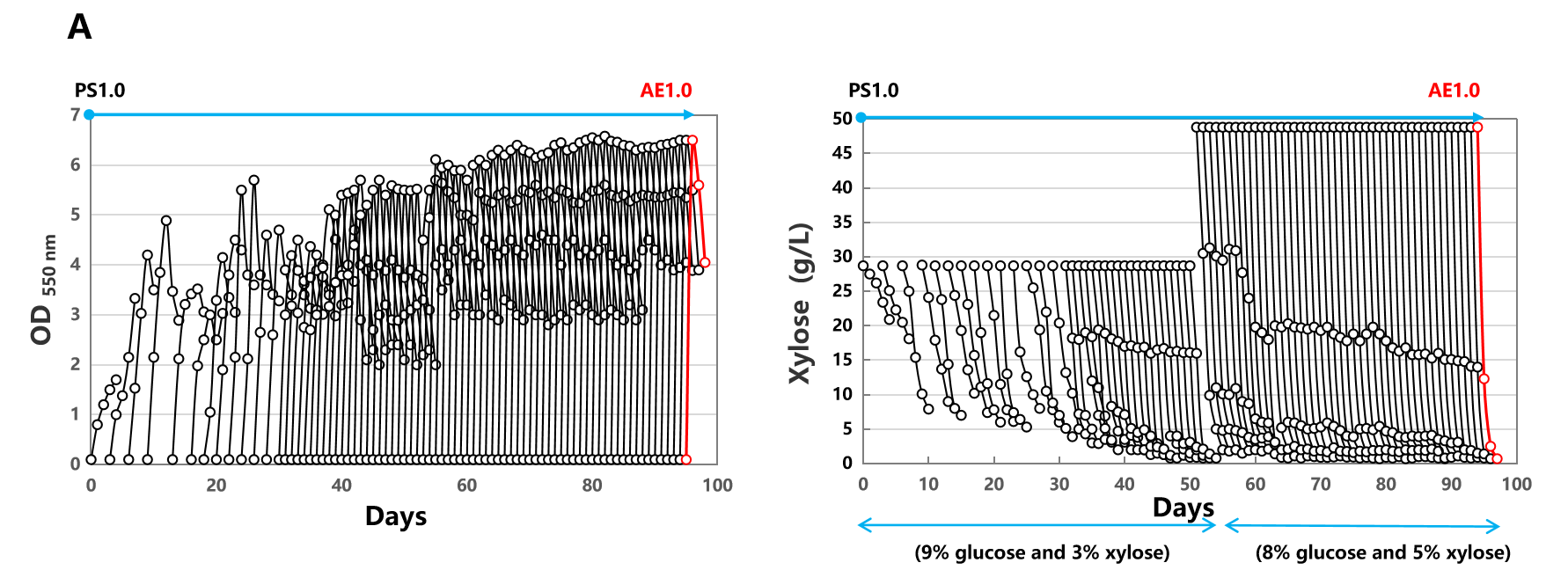


**
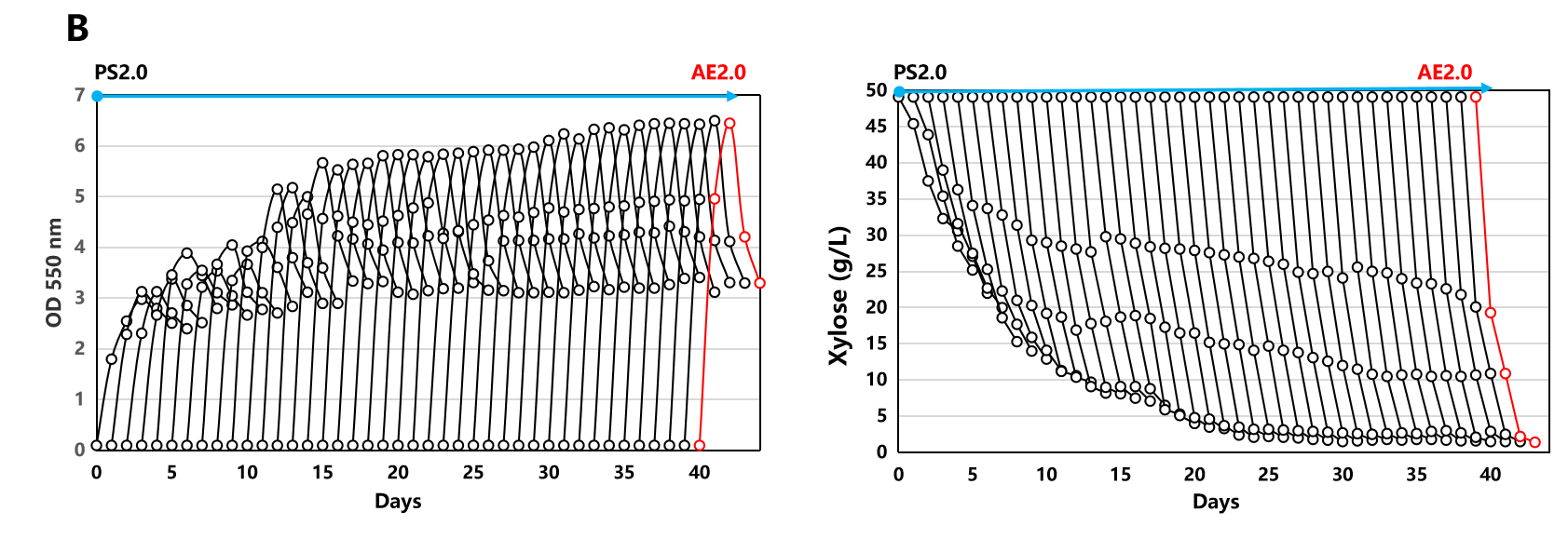
**

Fig. S2. New transporters were enriched and selected by adaptive evolution. (A) Starting strain PS1.0 was sequentially transferred in AM1 medium containing 9% glucose and 3% xylose at beginning 30 generation, and 8% glucose and 5% xylose after that for 50 generation. (B) Stating strain PS2.0 (AE1.0, ΔEcolC_1642*) was transferred in AM1 medium containing 8% glucose and 5% xylose for 38 generations. The red line indicates the culture used to isolate the strain AE1.0 and AE2.0.

**
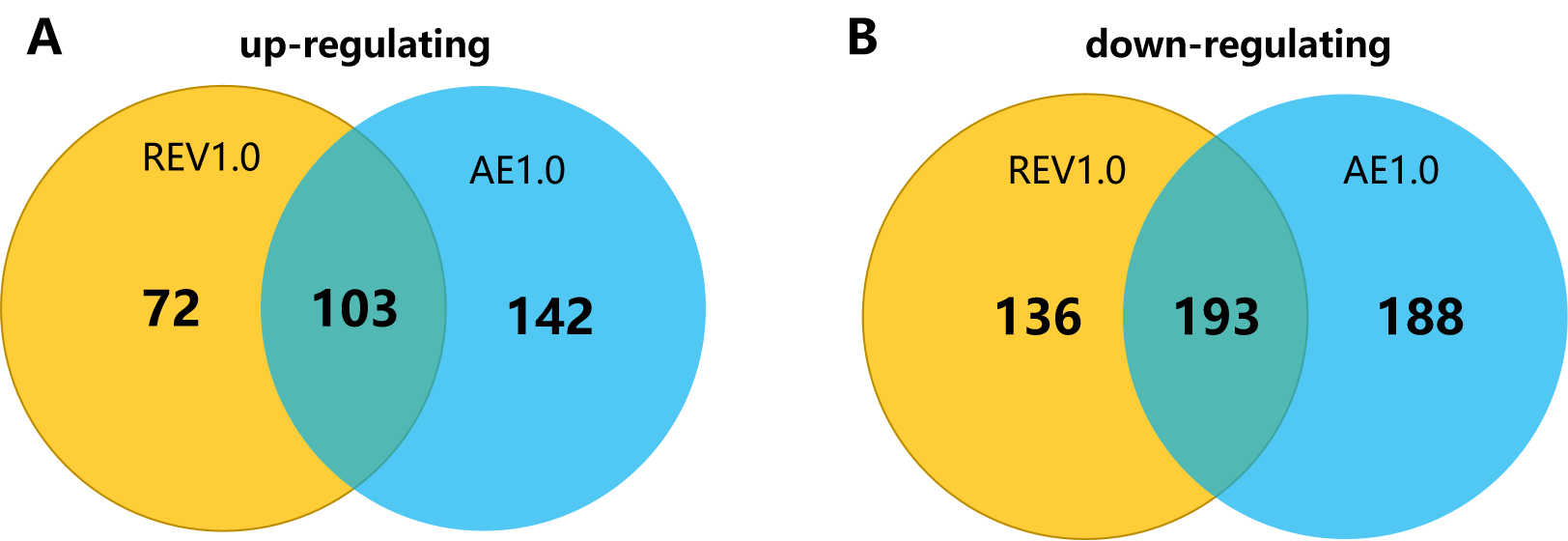
**

Fig. S3. Venn diagram of the transcriptome data in the strain REV1.0 (Precursor strain PS1.0 with *cyaA* gene deletion) and AE1.0 (Evolved strain PS1.0 with *cyaA* gene deletion), showing that cAMP-CRP involved in regulation of many genes expression.

**
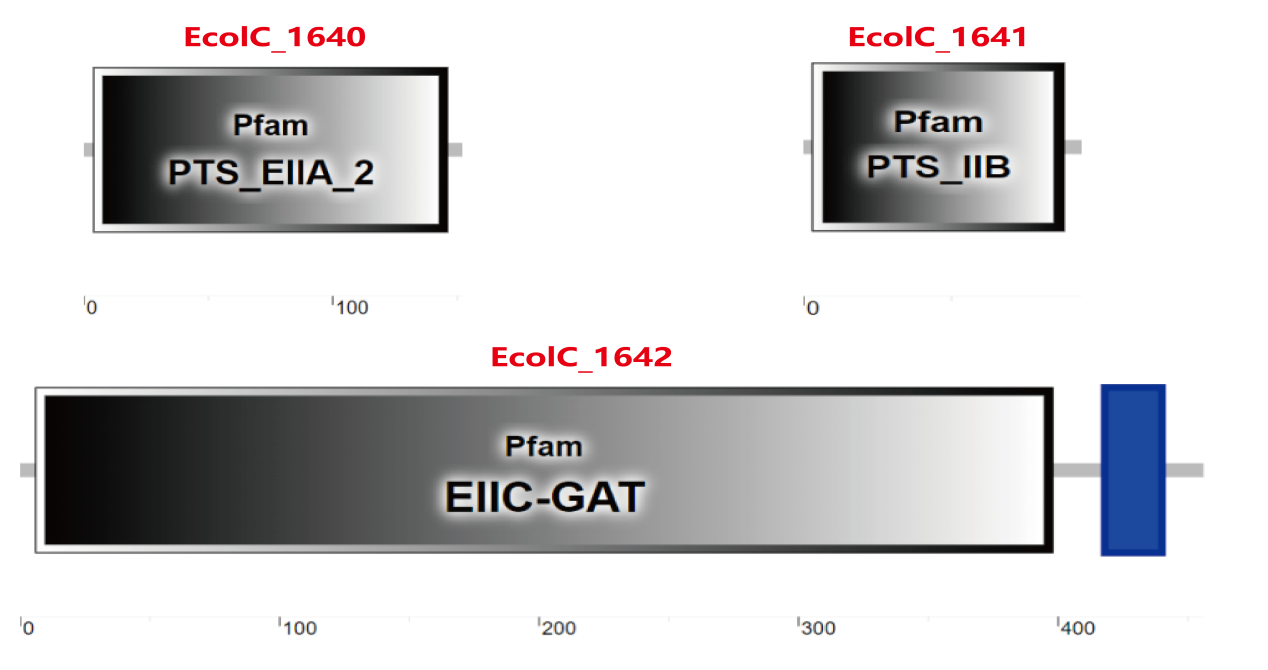
**

Fig. S4. Domain prediction of GalABC (encoded by *EcolC_1640, EcolC_1641 and EcolC_1642 genes*) using SMART online. These three proteins had typical enzyme EIIA-like (PF00359) (E-value, 4.4e-28), EIIB-like (PF02302) (E-value, 6.5e-13) and EIIC-like (PF03611) (E-value, 4.5e-60) domains, suggesting that GalABC is a PTS (phosphotransferase system) transporter.


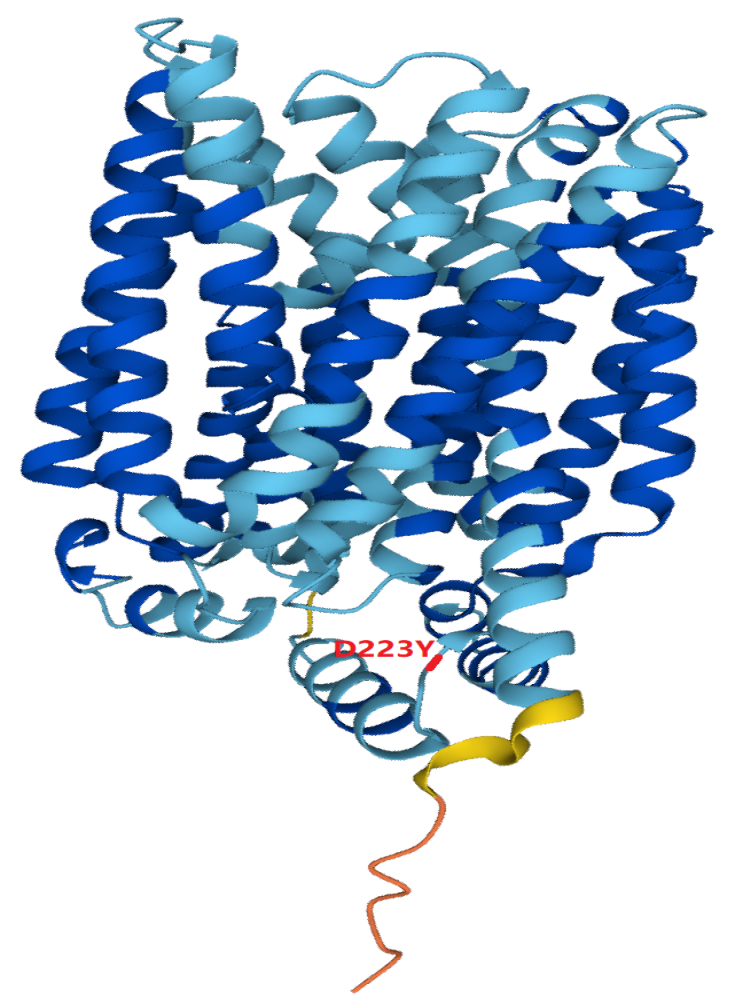


Fig. S5. The model structure of AraE in the UniProt online database (https://www.uniport.org/uniport/P0AE24). The D223Y (Red) position is located in the cytoplasmic loop between the 6th and 7th transmembrane helices.


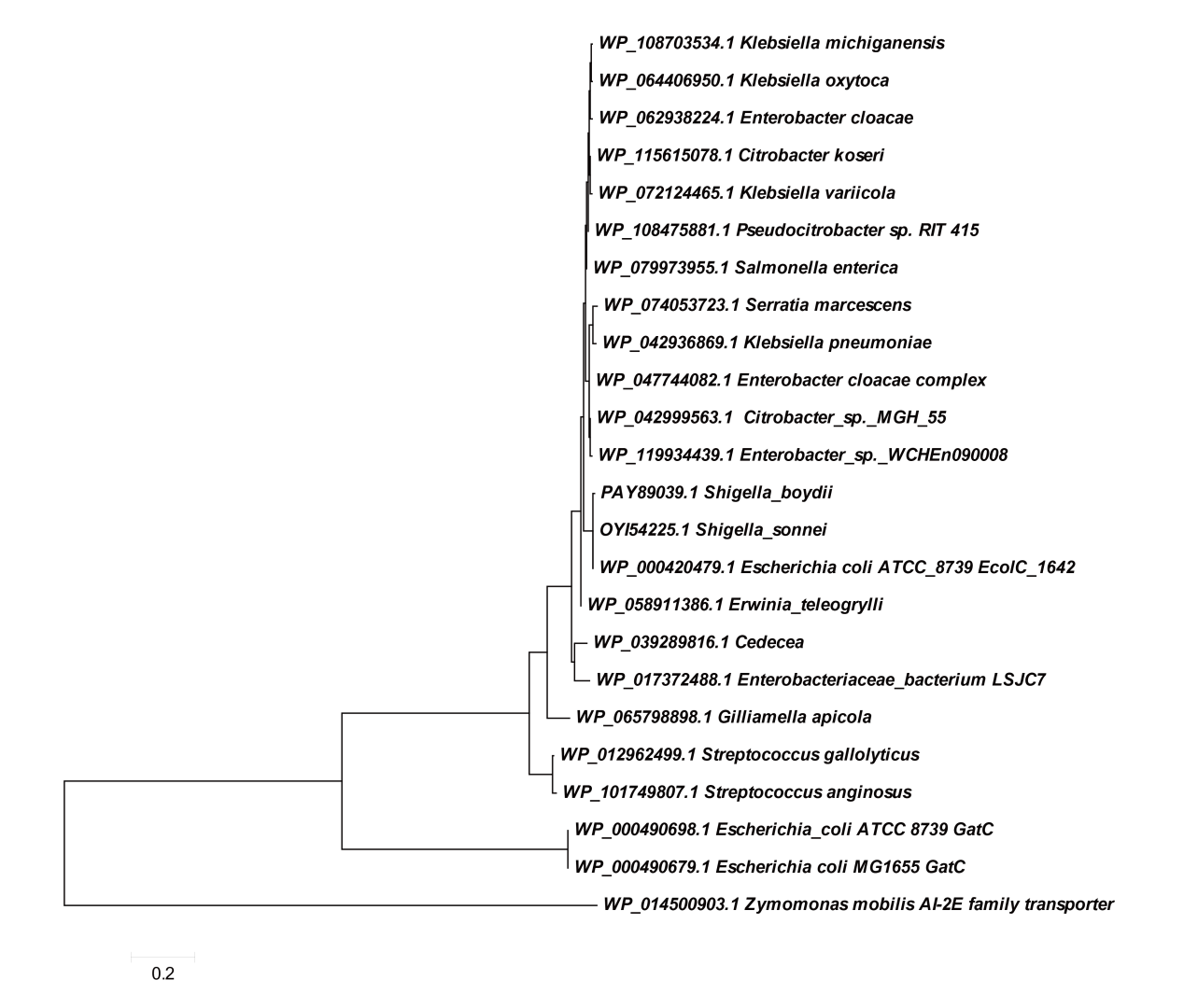


Fig. S6. Phylogenetic tree of EcolC_1642. The tree was constructed using Maximum Likelihood Tree approach.


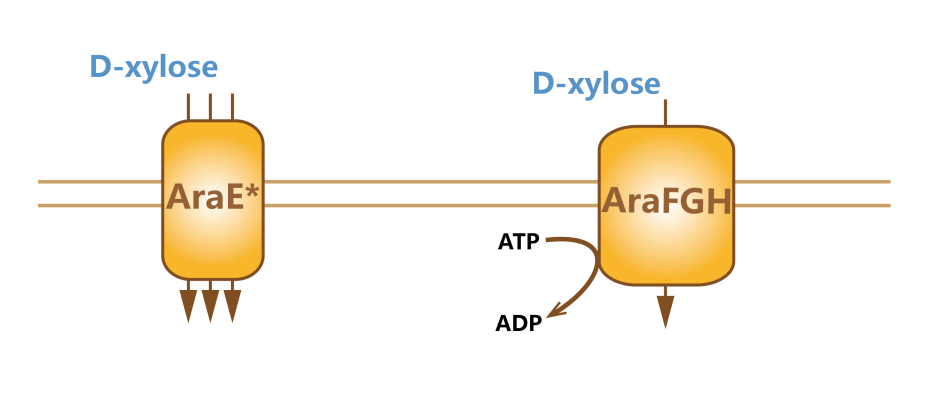


Fig. S7. Schematics of the contribution about AraE* and AraFGH on xylose transport. AraE* is the main xylose transporter, contributing 75% of the xylose flux through transportation, while AraFGH is the auxiliary transporter, contributing 25% to xylose flux.

**Table S**

**Talbe S1. Strains, plasmids, and primers used in this study**

| **Strains, plasmids, or primers** | **Relevant characteristics** | **Reference of source** |
| --- | --- | --- |
| **Strains** | | |
| HQ304 | Xylose utilization improved strain | Zhu et al., 2017 |
| PS1.0 | HQ304, Δ*zwf* and Δ*pgi* | This study |
| AE1.0 | PS1.0, first-round adaptively evolved strain | This study |
| Rec1.0 | AE1.0, recovering *zwf* and *pgi* | This study |
| HQ408 | PS1.0, introducing *EcolC_1642** mutation (N13S) | This study |
| REV1.0 | PS1.0, ∆*cyaA* | This study |
| HQ416 | PS1.0, introducing *EcolC_1642* mutation (N13S), and Δ*cyaA* | This study |
| HQ916 | AE1.0, recovering *EcolC_1642** mutation (N13S) | This study |
| HQ920 | AE1.0, recovering *cyaA* mutation | This study |
| HQ921 | AE1.0, recovering *EcolC_1642** and *cyaA* mutation to wild-type | This study |
| YM002 | REV1.0, ∆*EcolC_1642*::FRT-km-FRT | This study |
| YM004 | REV1.0, ∆*EcolC_1641*::FRT-km-FRT | This study |
| YM006 | REV1.0, ∆*EcolC_1640*::FRT-km-FRT | This study |
| PS2.0 | AE1.0, ∆*EcolC_1642**(N13S) | This study |
| AE2.0 | PS2.0, second-round adaptively evolved strain | This study |
| Rec2.0 | AE2.0, recovering *zwf* and *pgi* | This study |
| DL102 | PS2.0, introducing *araE**(D223Y) | This study |
| REV2.0 | PS2.0, introducing *araC**(L156I) | This study |
| DL104 | PS2.0, introducing *araE**and *araC** | This study |
| DL105 | REV2.0, ∆*araFGH*::FRT-km-FRT | This study |
| DL106 | REV2.0, ∆*araBDA*::FRT-km-FRT | This study |
| DL112 | AE2.0, ∆*araE** | This study |
| DL113 | AE2.0, ∆*araC** | This study |
| DL114 | AE2.0, ∆*araE** and ∆*araC** | This study |
| DL115 | AE2.0, ∆*araFGH* | This study |
| ATCC 8739 | *E. coli* wild type | Yomano et al., 2009 |
| ∆cyaA | ATCC 8739, ∆*cyaA* | This study |
| P_operon-wt_ | ∆*cyaA*, *P_operon_-lacZ* (the promoter of *lacZ* was replaced by the wild-type promoter-operator of *EcolC_1643* operon) | This study |
| P_CRP-mut_ | ∆*cyaA*, *P_operon-CRP_-lacZ* (the promoter of *lacZ* was replaced by the mutations in the cAMP-CRP element of promoter-operator of *EcolC_1643* operon) | This study |
| P_IHF-mut_ | ∆*cyaA*, *P_operon-IHF_-lacZ* (the promoter of *lacZ* was replaced by the mutations in the IHF element promoter-operator of *EcolC_1643* operon) | This study |
| P_CRP-IHF-mut_ | ∆*cyaA*, *P_operon-CRP-IHF_-lacZ* (the promoter of *lacZ* was replaced by the mutations in the cAMP-CRP and IHF elements promoter-operator of *EcolC_1643* operon) | This study |
| P100S | PS1.0, introducing *EcolC_1642* mutation (P100S) | This study |
| N124S | PS1.0, introducing *EcolC_1642* mutation (N124S) | This study |
| P100S/N13S | PS1.0, introducing *EcolC_1642* mutation (P100S/N13S) | This study |
| N124S/N13S | PS1.0, introducing *EcolC_1642* mutation (N124S/N13S) | This study |
| YM016 | PS1.0, introducing *gatC** (S184L) | This study |
| YM017 | YM016, M1-93-*gatABC** | This study |
| HQ409 | HQ408, M1-93-*EcolC_1642** | This study |
| **Plasmids** | | |
| pXZ-CS | cat-sacB cassette on pEASY Blunt vector | Tan et al., 2013 |
| pACYC184M | *cat*, p15A, replace *tet* with *lacI* and Ptrc of pTrc99AM | Zhao et al., 2013 |
| pRedCas9 | *kan*, P_BAD_-cas9, P_BAD_-Red-recA, repA101(Ts) | Zhu et al., 2017 |
| pRedCas9-serial | P_BAD_-cas9, P_BAD_-Red-recA,gRNA with constitutive promoter | Zhao et al., 2016 |
| placZ | *cat, P15A* from pACYC184-M, lacZ-N20-gRNA targeting *lacZ* gene expressed under constitute promoter, *lacZ* gene, its up and down homologous hand | Qiu et al., 2018 |
| pV4 | *cat*, p15A, Ptrc-gRNA-cat | This study |
| pV4-del-zwf | *cat*, p15A, Ptrc-gRNA-cat, Pcon-gRNA-zwf, Δ*zwf* | This study |
| pV4-del-pgi | *cat*, p15A, Ptrc-gRNA-cat, Pcon-gRNA-pgi, Δ*pgi* | This study |
| pV4-del-cyaA | *cat*, p15A, Ptrc-gRNA-cat, Pcon-gRNA-pgi, Δ*cyaA* | This study |
| pEASY-1642-WT | *EcolC_1642* wild-type in pEASY-blunt | This study |
| pEASY-1642-N13S | *EcolC_1642* mutation (N13S) in pEASY-blunt | This study |
| pEASY-1642-P100S | *EcolC_1642* mutation (P100S) in pEASY-blunt | This study |
| pEASY-1642-P124S | *EcolC_1642* mutation (P124S) in pEASY-blunt | This study |
| pEASY-1642-P100S/N13S | *EcolC_1642* mutation (P100S/N13S) in pEASY-blunt | This study |
| pEASY-1642-P124S/N13S | *EcolC_1642* mutation (P124S/N13S) in pEASY-blunt | This study |
| **Primers** | | |
| **Construction of pV4 plasmid** | | |
| cat-N20-up | CCAGGTCTCACGTCTGTGATGGCTTCCATGTGTTTTAGAGCTAGAAATAGCAAGTTAAAATAAGGC | This study |
| cat-N20-down | CCAGGTCTCAGCACCTGGCTAAATACGGAAGGATCT | This study |
| lacI-Ptrc-up | CCAGGTCTCATAGCGGCATGCATTTACGTTGACAC | This study |
| lacI-Ptrc-down | CCAGGTCTCAGACGTGTGAAATTGTTATCCGCTCA | This study |
| Bone-F | CCAGGTCTCAGTGCTTTTTCTTAGTCCGTGG | This study |
| Bone-R | CCAGGTCTCAGCTAACTACACGATGCTTTAACTGCAA | This study |
| **Construction of pV4-del-zwf** | | |
| V4-B-F1 | CCAGGTCTCAGTGCGCCATGAGAACGAACCATTG | This study |
| V4-B-R1 | CCAGGTCTCAGCTAAGATCTGACTCCATAACAGAGTACTCGC | This study |
| N20-B-R2 | CCAGGTCTCAACCGCTGGCTAAATACGGAAGGATCT | This study |
| zwf-N20-B-F2 | CCAGGTCTCATAGCCGAATACTTCGAGGAGTGCCGTTTTAGAGCTAGAAATAGCAAGTTAAAATAAGGC | This study |
| zwf-F1 | CCAGGTCTCACGGT TTATCGGGCGAAGCCAGAAT | This study |
| zwf-R1 | CCAGGTCTCACCCT TTCCAGTTGATACAGGGAAGG | This study |
| zwf-F2 | CCAGGTCTCAAGGG TTCCTGGAATGAGTTTGAGTAA | This study |
| zwf-R2 | CCAGGTCTCAGCAC GCCAACTGCGAACGATGAA | This study |
| zwf-YZ-up | TTCGCTAACATTGGCTTCC | This study |
| zwf-YZ-down | GGATAGTGTTCATAAGGCTGGT | This study |
| **Construction of pV4-del-pgi** | | |
| pgi-N20-B-F2 | CCAGGTCTCATAGCAATCACTCCGTTCAGCCTGGGTTTTAGAGCTAGAAATAGCAAGTTAAAATAAGGC | This study |
| pgi-F1 | CCAGGTCTCACGGT TGCGAACGCTTCTGGTGA | This study |
| pgi-R1 | CCAGGTCTCACCCTGTCTGCGTTGGATTGATGTTT | This study |
| pgi-F2 | CCAGGTCTCAAGGGAGCCACGATAGCTCGACCAA | This study |
| pgi-R2 | CCAGGTCTCAGCACTTCCGTTAAATCACAGACAAGG | This study |
| pgi-YZ-UP | CGTTGGCATCAGAAAGCACA | This study |
| pgi-YZ-down | GGGAATTAGCGATGGTGTTT | This study |
| **Construction of pV4-del-cyaA** | | |
| cyaA-N20-B-F2 | CCAGGTCTCATAGCGCAAAGTTGTTCCTCTGACTGTTTTAGAGCTAGAAATAGCAAGTTAAAATAAGGC | This study |
| cyaA-F1 | CCAGGTCTCACGGTGCTCGCCATCAACTTGTCTT | This study |
| cyaA-R1 | CCAGGTCTCACCCTCACGCAATTGATTTATGGCA | This study |
| cyaA-F2 | CCAGGTCTCA AGGG GTGAAGGTTGATGGTCGTGAA | This study |
| cyaA-R2 | CCAGGTCTCA GCACAAACTTTGAAGCCCTGACCC | This study |
| cyaA-YZ-up | GAATGTACGGCGATATCGGC | This study |
| cyaA-YZ-down | AAAGGCGTGGTGTAACTGC | This study |
| **Deletion of *EcolC_1642* gene** | |  |
| 1642-FRT-up | CCGCTGATGTACGCTGGAATATGGGTTTCCGACCAGATGATGAAAAATCTGAGGGTTAATGTATGGTGTAGGCTGGAGCTGCTTC | This study |
| 1642-FRT-down | GAATAATGATAGCGCCCATGCTGGAGCCAAATCCCATAACCGCAGTCGCTGCGCCACCAACATATGGGAATTAGCCATGGTCC | This study |
| **Deletion of *EcolC_1641* gene** | |  |
| 1641-FRT-up | GCCAGATTGAAACGGATTTGGAAAAAGCGCTTTAAATCTTTTACAGAGGAATAATCAAATGGTGTAGGCTGGAGCTGCTTC | This study |
| 1641-FRT-down | CACTGATGCTGTTATCAACATCATTCACCAGAATTTCAAATTTCATTGCACACCTGTTAATGGGAATTAGCCATGGTCC | This study |
| **Deletion of *EcolC_1640* gene** | | |
| 1640-FRT-up | GCGAAGATGAGATTTTCGCTGCAGTTAAAGAAGAGATGTTGAAATAACAGGTGTGCAATGGTGTAGGCTGGAGCTGCTTC | This study |
| 1640-FRT-down | GATTAATAGCCATATCAGTGAAGAAAGGGCAGTACCGCTGATTCTGCCCTTTTAATTTCAATGGGAATTAGCCATGGTCC | This study |
| **Deletion of *araFGH* gene** | | |
| araFGH-FRT-up | TTTACCGAAGTTACCGACGTGGTACTGATCACGCGTGACAACTTTAAAGAAGAACTGGGATGGTGTAGGCTGGAGCTGCTTC | This study |
| araFGH-FRT-down | AGATACGCCCGAAGCTGAATGACGACTTAGGTGCGCCAGACCCCGATGTAGAAACAGAAGAATGGGAATTAGCCATGGTCC | This study |
| **Deletion of *araBDA* gene** | | |
| araBDA-FRT-up | CCGGAGTTTGCCGAAAACCCGAACGCGATGTTCGTATTGTGGAAAGACCACACTGCGGTATGGTGTAGGCTGGAGCTGCTTC | This study |
| araBDA-FRT-down | GACCGCCACTTCACGCATGTTATCGCCAAAACGGCAGACTTTCAGATGACGGGTATCCTGATGGGAATTAGCCATGGTCC | This study |
| **Integration or restoring of** *EcolC_1642******** | | |
| 1642*-cat-up | CCGCTGATGTACGCTGGAATATGGGTTTCCGACCAGATGATGAAAAATCTGAGGGTTAATGTATGTGTGACGGAAGATCACTTCGCA | This study |
| 1642*-sacB-down | GAATAATGATAGCGCCCATGCTGGAGCCAAATCCCATAACCGCAGTCGCTGCGCCACCAACATTTATTTGTTAACTGTTAATTGTCCT | This study |
| 1642-up | CGACTCAGCAATAACGCACAA | This study |
| 1642-down | TCGCCGAGAATGCCAAAC | This study |
| **Integration of gat*C**** | | |
| gatC*-cat-up | TATGACGCGGGTGGTAAATGTTGATATCTGGAATATCTGGCATATGACCTTCACCGTGTGACGGAAGATCACTTCGCA | This study |
| gatC*-sacB-down | CGAGCCTGCTTAGCGATGGGCGTTAAACCATCCATGATGGGTTTAATCACCCGTGGCATTATTTGTTAACTGTTAATTGTCC | This study |
| gatC-F1 | TTGGCTTTGTTGGCATTG | This study |
| gatC*-R1 | ATACGCCAGCGTACCGTG | This study |
| gatC*-F2 | GGTACGCTGGCGTATATGG | This study |
| gatC-R2 | AACGGCAGCACCTGATTAC | This study |
| **Modulation of *EcolC_1642** operon** | | |
| 1642*-cat-up | CCGCTGATGTACGCTGGAATATGGGTTTCCGACCAGATGATGAAAAATCTGAGGGTTAATGTGACGGAAGATCACTTCGCA | This study |
| 1642*-sacB-down | GAAAGATGACCGGGATAAATATAAAATTTCCCAGATCGTTTATAAATTGCGTGATCAGTTCCATTTATTTGTTAACTGTTAATTGTCCT | This study |
| 1642*-P-up | CCGCTGATGTACGCTGGAATATGGGTTTCCGACCAGATGATGAAAAATCTGAGGGTTAATTATCTCTGGCGGTGTTGAC | This study |
| 1642*-RBS-down | AGATGACCGGGATAAATATAAAATTTCCCAGATCGTTTATAAATTGCGTGATCAGTTCCATAGCTGTTTCCTGGTTTAAACGTACATG | This study |
| **Modulation of *gatABC** operon** | | |
| gatABC*-cat-up | GCTGATTATGGATAAAATTTATGATGTTTTGCGCGCCTATCGCTACGGCTGTGCGGAATAATGTGACGGAAGATCACTTCGCA | This study |
| gatABC*-sacB-down | GGGTTAAAACTTCGCTACGATCGACAAAAGAAATTCCGCTACGAACAAACAGGTTAGTCATTTATTTGTTAACTGTTAATTGTCCT | This study |
| gatABC*-P-up | GCTGATTATGGATAAAATTTATGATGTTTTGCGCGCCTATCGCTACGGCTGTGCGGAATAATTATCTCTGGCGGTGTTGAC | This study |
| gatABC*-RBS-down | GGGTTAAAACTTCGCTACGATCGACAAAAGAAATTCCGCTACGAACAAACAGGTTAGTCATAGCTGTTTCCTGGTTTAAACGTACATG | This study |
| **P_operon_ mutations** | | |
| P_operon_-up | AATGATTTTTTATTATTCATTACAACGCCAC | This study |
| P_operon_-down | TTCATTTTGCCTGTCGTTAAGTAACT | This study |
| CRP-mut-up | CCTGATTCAGATCTCAATTGACAACCA | This study |
| CRP-mut-down | AGATCTGAATCAGGAAATTTATAAGTAATTGATTTTTATG | This study |
| IHF-mut-up | GTTTACTTATAAATTTTGTGATTCAGATCTCA | This study |
| IHF-mut-down | ATTTATAAGTAAACGATTTTTATGGTATTAAAAACC | This study |
| CRP-IHF-mut-up | CCTGATTCAGATCTCAATTGACAACCA | This study |
| CRP-IHF-mut-up | AGATCTGAATCAGGAAATTTATAAGTAAACGATTTTTATG | This study |
| ***EcolC_1642* mutations** | | |
| 1642-CX-up | CGACTCAGCAATAACGCACAA | This study |
| 1642-CX-down | TCGCCGAGAATGCCAAAC | This study |
| P100S-1642-TB-F | TCCCTCTGTGTTGCGGTAAATATTGC | This study |
| P100S-1642-TB-R | ACACAGAGGGAAATAATGATAGCGCCCATG | This study |
| N124S-1642-TB-F | TCCCTTCATCAAAATGCGTCAATGG | This study |
| N124S-1642-TB-R | TTGATGAAGGGAGAAAACATCAACGTTAAC | This study |

**Table S2 Comparison of sugar utilization by representative engineered *E. coli* strains**

| **Strains^a^** | **Genetic modifications** | **Utilization rate**  **(g/gDWC·h)** | | **q_gluc/_q_xyl_**  **(mol/mol)** | **Specific growth rate (h^-1^)** | **Cell mass**  **(g/L)** |
| --- | --- | --- | --- | --- | --- | --- |
|  |  | **Glucose** | **Xylose** |  |  |  |
| HQ304 | - | 0.69 ±0.02 | 0.35 ±0.02 | 1.64 | 0.17 ±0.01 | 1.31±0.01 |
| PS1.0 | HQ304, Δ*zwf*, Δ*pgi* | 0 | 0.1±0.01 | - | 0.05±0.00 | 0.41±0.00 |
| AE1.0 | PS1.0, Evolved strain | 0.02±0.003 | 0.8±0.01 | - | 0.25 ±0.01 | 1.98±0.01 |
| Rec1.0 | AE1.0, recovering *zwf* and *pgi* | 0.71 ±0.03 | 0.63 ±0.03 | 0.94 | 0.26 ±0.01 | 2.05±0.01 |
| PS2.0 | AE1.0, Δ*EcolC_1642** | 0 | 0.16±0.01 | - | 0.023±0.01 | 0.18±0.01 |
| AE2.0 | PS2.0, evolved strain | 0.02±0.001 | 0.82±0.02 | - | 0.21±0.01 | 1.63±0.01 |
| Rec2.0 | HQ302, recovering *zwf* and *pgi* | 0.76±0.02 | 0.66±0.01 | 0.95 | 0.23 ±0.01 | 1.82±0.02 |

**^a^** Fermentations were performed in AM1 with 5% glucose and 5% xlyose.

**Table S3 Transcriptome results of adaptively evolved strains AE1.0 and AE2.0**

| **Gene** | **Name** | **Product** | **PS1.0 FPKM** | **AE1.0 FPKM** | **PS2.0 FPKM** | **AE2.0 FPKM** | **AE1.0/PS1.0** | **AE2.0/PS2.0** |
| --- | --- | --- | --- | --- | --- | --- | --- | --- |
| EcolC_4201 | *cyaA* | adenylate cyclase | 97 | 0 | 0 | 0 | 0 | 0 |
| EcolC_1643 | - | ribose/galactose 5-phosphate isomerase | 26 | 1842 | 7303 | 1155 | 71.25 | 0.16 |
| EcolC_1642 | - | PTS galactitol transporter subunit IIC | 30 | 1794 | 179 | 47 | 59.72 | 0.26 |
| EcolC_1641 | - | PTS galactitol transporter subunit IIB | 21 | 1420 | 35 | 2 | 67.26 | 0.06 |
| EcolC_1640 | - | PTS galactitol transporter subunit IIA | 39 | 1939 | 82 | 24 | 50.16 | 0.29 |
| EcolC_0874 | *araE* | arabinose-proton symporter | 95 | 47 | 24 | 1378 | 0.49 | 58.38 |
| EcolC_1734 | *araF* | L-arabinose-binding periplasmic protein | 627 | 10 | 192 | 451 | 0.02 | 2.35 |
| EcolC_1735 | *araG* | arabinose import ATP-binding protein AraG | 339 | 14 | 34 | 297 | 0.04 | 8.79 |
| EcolC_1736 | *araH* | arabinose ABC transporter permease | 585 | 23 | 56 | 217 | 0.04 | 3.90 |
| EcolC_3593 | *araC* | Arabinose operon regulatory protein | 117 | 40 | 107 | 357 | 0.34 | 3.34 |
| EcolC_3594 | *araB* | Ribulokinase | 163 | 50 | 30 | 6097 | 0.31 | 200.29 |
| EcolC_3595 | *araA* | L-arabinose isomeraes | 334 | 69 | 32 | 5704 | 0.21 | 180.72 |
| EcolC_3596 | *araD* | L-ribulose-5-phosphate-4-epimerase | 283 | 54 | 23 | 5788 | 0.19 | 249.89 |

FPKM: Fragments Per Kilobase per Million.

**Table S4 The effects of *EcolC_1642*, *EcolC_1641*, *EcolC_1640*, *araFGH* and *araBDA* gene deletion on xylose utilization**

| Strain | Xylose utilization rate (g/gDCW·h) | Specific growth rate (h^-1^) | Cell mass  (g/L) |
| --- | --- | --- | --- |
| REV1.0 (PS1.0, ∆*cyaA*) | 0.54±0.02 | 0.17±0.02 | 1.38±0.02 |
| REV1.0, ∆1642*::FRT-km-FRT* | 0.11±0.02 | 0.04±0.01 | 0.43±0.01 |
| REV1.0, ∆1641*::FRT-km-FRT* | 0.12±0.01 | 0.04±0.01 | 0.42±0.00 |
| REV1.0, ∆1640*::FRT-km-FRT* | 0.12±0.01 | 0.04±0.01 | 0.46±0.00 |
| REV2.0 (PS2.0, introducing *araC**(L156I)) | 0.51±0.01 | 0.18±0.01 | 1.42±0.02 |
| REV2.0, ∆*araFGH::FRT-km-FRT* | 0.39±0.02 | 0.14±0.00 | 1.08±0.01 |
| REV2.0, ∆*araBDA::FRT-km-FRT* | 0.52±0.01 | 0.18±0.01 | 1.45±0.01 |

**Table S5 Comparison of glucose and xylose co-utilization capabilities of engineered *E. coli* strains**

| Strain | Genetics | Culture | Utilization rate (g/gDCW·h) | | q_gluc_/q_xyl_  (mol/mol) | References |
| --- | --- | --- | --- | --- | --- | --- |
|  |  |  | Glucose | Xylose |  |  |
| IT1168 | Δ*ptsG* | Anaerobic, LB  4% glucose, 4% xylose | 0.21 | 0.28 | 0.63 | Nichols et al., 2001 |
| W3110C | Δ*ccr* | Anaerobic, AM1  2% glucose, 2% xylose | 0.37 | 0.38 | 0.81 | Liang et al., 2015 |
| PC05^a^ | Crp* | Aerobic, LB  2% glucose, 2% xylose | 0.18 | 0.4 | 0.38 | Cirino et al., 2006 |
| KO11  LY168^a^ | Δ*mgsA* | Anaerobic, AM1  5% glucose, 5% xylose | 0.75 | 0.38 | 1.64 | Yamano et al., 2009 |
| AS1600^a^ | GalP* | Anaerobic, AM1  5% glucose, 5% xylose | 0.52 | 0.40 | 1.08 | Sawisit et al., 2015 |
| KO11  XW043 | XylR* | Anaerobic, AM1  5% glucose, 5% xylose | 0.69 | 0.57 | 1.01 | Sievert et al., 2017 |
| Rec1.0 | EcolC_1642* | Anaerobic, AM1  5% glucose, 5% xylose | 0.71 | 0.63 | 0.94 | This work |
| Rec2.0 | AraE* | Anaerobic, AM1  5% glucose, 5% xylose | 0.76 | 0.66 | 0.95 | This work |

^a^The utilization rate of glucose and xylose were calculated by the data shown up in their context.

**Table S6 Xylose utilization by *E. coli* strains having *gatABC** and *EcolC_1642** genes modulated by artificial promoter**

| **Strain** | **Genetics** | **Xylose utilization rate (g/gDWC/h)** | **Specific growth rate (h^-1^)** | **Cell mass(g/L)** |
| --- | --- | --- | --- | --- |
| YM016 | PS1.0, introducing *gatC** (S184L) | 0.12±0.02 | 0.02±0.00 | 0.17±0.02 |
| YM017 | YM016, M1-93-*gatABC** | 0.11±0.01 | 0.02±0.00 | 0.17±0.01 |
| HQ408 | PS1.0, introducing *EcolC_1642** (N13S) | 0.31±0.04 | 0.09±0.01 | 0.73±0.01 |
| HQ409 | HQ408, M1-93-*EcolC_1642** | 0.54±0.02 | 0.13±0.01 | 1.03±0.02 |

**Table S7 The effect of *EcolC_1642* mutations on xylose utilization**

| Strain | Genetics | Xylose utilization rate (g/gDCW·h) | Specific growth rate (h-1) | Cell mass  (g/L) |
| --- | --- | --- | --- | --- |
| PS1.0 | - | 0.10±0.02 | 0.02±0.00 | 0.14±0.00 |
| HQ408 | PS1.0, *EcolC_1642* (N13S) | 0.31±0.04 | 0.09±0.01 | 0.73±0.01 |
| P100S | PS1.0, *EcolC_1642* (P100S) | 0.27±0.02 | 0.01±0.01 | 0.76±0.02 |
| N124S | PS1.0, *EcolC_1642* (N124S) | 0.23±0.03 | 0.09±0.01 | 0.72±0.01 |
| P100S/N13S | PS1.0, *EcolC_1642* (P100S /N13S) | 0.36±0.03 | 0.09±0.01 | 0.73±0.02 |
| N124S/N13S | PS1.0, *EcolC_1642* (N124S/N13S) | 0.33±0.02 | 0.09±0.01 | 0.69±0.01 |

**Table S8 Other gene expression in CyaA inactivate strain**

| **Gene** | **Name** | **Product** | **PS1.0**  **FPKM** | **AE1.0 FPKM** | **AE2.0 FPKM** | **AE1.0/PS1.0** | **AE2.0/PS1.0** |
| --- | --- | --- | --- | --- | --- | --- | --- |
| **Glycolysis pathway** | | | | | | | |
| EcolC_0771 | *galP* | galactose-proton symporter | 20727 | 10681 | 7747 | 0.52 | 0.37 |
| EcolC_1281 | *glk* | glucokinase | 28784 | 484 | 182 | 0.02 | 0.01 |
| EcolC_4102 | *pfkA* | 6-phosphofructokinase | 334 | 702 | 468 | 2.10 | 1.40 |
| EcolC_0785 | *fbaA* | fructose bisphosphate aldolase class I I | 1482 | 3605 | 3519 | 2.43 | 2.37 |
| EcolC_4099 | *tpiA* | triose phosphate isomerase | 672 | 1792 | 1338 | 2.67 | 1.99 |
| EcolC_1853 | *gapA* | glyceraldehyde 3-phosphate dehydrogenase A | 2957 | 14300 | 20023 | 4.84 | 6.77 |
| EcolC_0784 | *pgk* | phosphoglycerate kinase | 816 | 1547 | 1240 | 1.90 | 1.52 |
| EcolC_2907 | *gpmA* | phosphoglycerate mutase 1 | 760 | 764 | 680 | 1.01 | 0.89 |
| EcolC_0933 | *eno* | enolase | 499 | 4727 | 4046 | 9.47 | 8.11 |
| EcolC_4005 | *pgi* | glucosephosphate isomerase | 69 | 230 | 421 | 3.33 | 6.10 |
| EcolC_1778 | *pykF* | pyruvate kinase II | 648 | 1203 | 1818 | 1.86 | 2.81 |
| EcolC_1955 | *pykF* | pyruvate kinase I | 153 | 514 | 401 | 3.36 | 2.62 |
| EcolC_4060 | *ppc* | phosphoenolpyruvate carboxylase | 307 | 1487 | 994 | 4.85 | 3.24 |
| **The metabolism of sugars related to cAMP-CRP** | | | | | | | |
| EcolC_3281 | *lacZ* | beta-galactosidase | 6 | 2 | 2 | 0.43 | 0.34 |
| EcolC_3282 | *lacY* | lactose permease | 7 | 1 | 1 | 0.18 | 0.18 |
| EcolC_3283 | *lacA* | galactoside O-acetyltransferase | 3 | 2 | 0 | 0.72 | 0.00 |
| *EcolC_1551* | *gatY* | tagatose-1,6-bisphosphate aldolase | 4 | 1 | 2 | 0.14 | 0.51 |
| EcolC_1552 | *gatZ* | tagatose-1,6-bisphosphate aldolase | 4 | 2 | 0 | 0.44 | 0.12 |
| EcolC_1553 | *gatA* | galactitol-specific phosphotransferase enzyme IIA component | 1 | 0 | 0 | 0.00 | 0.00 |
| EcolC_1554 | *gatB* | galactitol-specific phosphotransferase enzyme IIB component | 0 | 0 | 0 | 0.00 | 0.00 |
| EcolC_1555 | *gatC* | PTS galactitol transporter subunit IIC | 10 | 4 | 4 | 0.43 | 0.36 |
| EcolC_1556 | *gtaD* | galactitol-1-phosphate 5-dehydrogenase | 8 | 3 | 2 | 0.39 | 0.20 |
| EcolC_3994 | *malK* | maltose/maltodextrin import ATP-binding protein MalK | 525 | 10 | 7 | 0.02 | 0.01 |
| EcolC_3995 | *malE* | maltose-binding periplasmic protein | 1788 | 69 | 37 | 0.04 | 0.02 |
| EcolC_3996 | *malF* | maltose ABC transporter permease | 465 | 30 | 9 | 0.06 | 0.02 |
| EcolC_3997 | *malG* | maltose ABC transporter permease | 1036 | 39 | 20 | 0.04 | 0.02 |
| EcolC_3993 | *lamB* | maltoporin | 854 | 27 | 8 | 0.03 | 0.01 |
| EcolC_3992 | *malM* | maltose operon protein | 427 | 32 | 14 | 0.07 | 0.03 |
| EcolC_2017 | *manA* | mannose-6-phosphate isomerase | 162 | 228 | 130 | 1.41 | 0.80 |

FPKM: Fragments Per Kilobase per Million.

**References**

Engler C, Kandzia R, Marillonnet S (2008) A one pot, one step, precision cloning method with high throughput capability. PLoS One 3, e3647.

Chen J, Zhu X, Tan Z, Xu H, Tang J, Xiao D, Zhang X (2014) Activating C4-dicarboxylate transporters DcuB and DcuC for improving succinate production. *Appl Microbiol Biotechnol* 98 (5):2197-2205. doi:10.1007/s00253-013-5387-7.

Cirino PC, Chin JW, Ingram LO (2006) Engineering *Escherichia coli* for xylitol production from glucose-xylose mixtures. *Biotechnol Bioeng* 95(6):1167-1176.

Lu J, Tang J, Liu Y, Zhu X, Zhang T, Zhang X (2012) Combinatorial modulation of *galP* and *glk* gene expression for improved alternative glucose utilization. *Appl Microbiol Biotechnol* 93(6):2455-2462.

Nichols NN, Dien BS, Bothast RJ (2001) Use of catabolite repression mutants for fermentation of sugar mixtures to ethanol. *Appl Microbiol Biotechnol* 56(1-2):120-125.

Qiu H, Zhao D, Man S, Bi C, Zhu X, Zhang X (2018) Construction of promoter with tight regulation on chromosome of *Eshcherichia coli*. *Microbiol. China*. 45, 1693-1704.

Tan Z, Zhu X, Chen J, Li Q, Zhang X. (2013) Activating phosphoenolpyruvate carboxylase and phosphoenolpyruvate carboxykinase in combination for improvement of succinate production. *Appl Environ Microb* 79(16):4838-4844.

Sawisit A, Jantama K, Zheng H, Yomano LP, York SW, Shanmugam KT, Ingram LO (2015) Mutation in *galP* improved fermentation of mixed sugars to succinate using engineered *Escherichia coli* AS1600a and AM1 mineral salts medium. *Bioresour Technol* 193:433-441.

Sievert C, Nieves LM, Panyon LA, Loeffler T, Morris C, Cartwright RA, Wang X (2017) Experimental evolution reveals an effective avenue to release catabolite repression via mutations in XylR. *Proc Natl Acad Sci* U S A 114(28):7349-7354.

Yomano LP, York SW, Shanmugam KT, Ingram LO (2009) Deletion of methylglyoxal synthase gene (*mgsA*) increased sugar co-metabolism in ethanol-producing *Escherichia coli*. *Biotechnol Lett* 31(9):1389-1398.

Zhao D, Yuan S, Xiong B, Sun H, Ye L, Li J, Zhang X, Bi C (2016) Development of a fast and easy method for *Escherichia coli* genome editing with CRISPR/Cas9. *Microbial Cell Factories* 15 (1):205. doi:10.1186/s12934-016-0605-5.

Zhao J, Li Q, Sun T, Zhu X, Xu H, Tang J, Zhang X, Ma Y. (2013) Engineering central metabolic modules of *Escherichia coli* for improving beta-carotene production. *Metab Eng* 17:42-50.

Zhu X, Tan Z, Xu H, Chen J, Tang J, Zhang X (2014) Metabolic evolution of two reducing equivalent-conserving pathways for high-yield succinate production in *Escherichia coli*. *Metab Eng* 24:87-96. doi:10.1016/j.ymben.2014.05.003

Zhu X, Zhao D, Qiu H, Fan F, Man S, Bi C, Zhang X (2017) The CRISPR/Cas9-facilitated multiplex pathway optimization (CFPO) technique and its application to improve the *Escherichia coli* xylose utilization pathway. *Metab Eng* 43 (Pt A):37-45. doi:10.1016/j.ymben.2017.08.003.
